# Supplementary material for: Transcriptional effects of copy number alterations in a large set of human cancers
Source: Nat Commun. 2020 Feb 5;11:715. doi: 10.1038/s41467-020-14605-5 (PMC7002723; doi:10.1038/s41467-020-14605-5)
Supplement: Supplementary file 2 — Description of Additional Supplementary Files [file 41467_2020_14605_MOESM2_ESM.pdf]

Description of Additional Supplementary Files

File Name: Supplementary Data 1 -
Description: Tissues types represented in the GEO dataset

File Name: Supplementary Data 2
Description: Tissues types represented in the TCGA dataset

File Name: Supplementary Data 3
Description: Tissues types represented in the CCLE dataset

File Name: Supplementary Data 4
Description: Tissues types represented in the GDSC dataset

File Name: Supplementary Data 5
Description: Genes located in a marked genomic region of at least one CNA-CES

File Name: Supplementary Data 6
Description: Colocalization of borders of marked genomic regions of CNA-CESs with common fragile sites

File Name: Supplementary Data 7
Description: Correlations between the weight of genes in CNA-CESs across datasets

File Name: Supplementary Data 8
Description: Correlations between GSEA results in the GEO-dataset and TCGA-dataset

File Name: Supplementary Data 9
Description: Gene set enrichment analysis per cytoband on the degree of transcriptional adaptation*

File Name: Supplementary Data 10
Description:Gene set enrichment analysis on TACNA expression levels in the TCGA dataset using the protein complex geneset database from CORUM*

File Name: Supplementary Data 11
Description: Significance of pearson correlation coefficients between TACNA expression levels of genes from protein complex subunits

File Name: Supplementary Data 12
Description: Presence of individual genes in marked genomic regions of CNA-CESs in the GEO and TCGA datasets

File Name: Supplementary Data 13
Description: Average degree of transcriptional adaptation of oncogenes

File Name: Supplementary Data 14
Description: Spearman correlations between average TACNA profiles per tumor type

File Name: Supplementary Data 15

Description: Correlations between inferred CNA burden and expression-based immune metric per tumor type

File Name: Supplementary Data 16

Description: Spearman correlations between TACNA expression levels and inferred immune cell type abundance

File Name: Supplementary Data 17

Description: Gene set enrichment analysis on correlations between TACNA expression levels and inferred CD8+ T cell abundance
