# Supplementary material for: Transcriptional effects of copy number alterations in a large set of human cancers
Source: Nat Commun. 2020 Feb 5;11:715. doi: 10.1038/s41467-020-14605-5 (PMC7002723; doi:10.1038/s41467-020-14605-5)
Supplement: Supplementary file 20 — Supplementary Information [file 41467_2020_14605_MOESM20_ESM.pdf]

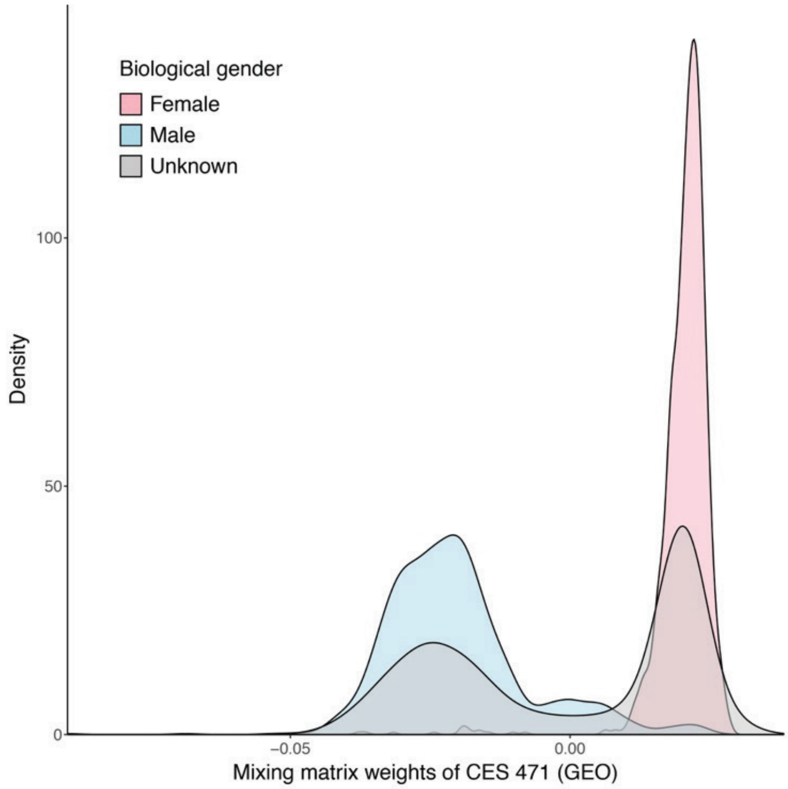

Supplementary Fig. 1 Gender CES. A density plot is shown for the mixing matrix weights of CES 471 in the GEO dataset. Males (*n* = 1,074), females (*n* = 1,300) and unknown biological gender status (*n* = 19,218) is plotted separately depending on the available information provided online by the original authors (GEO).

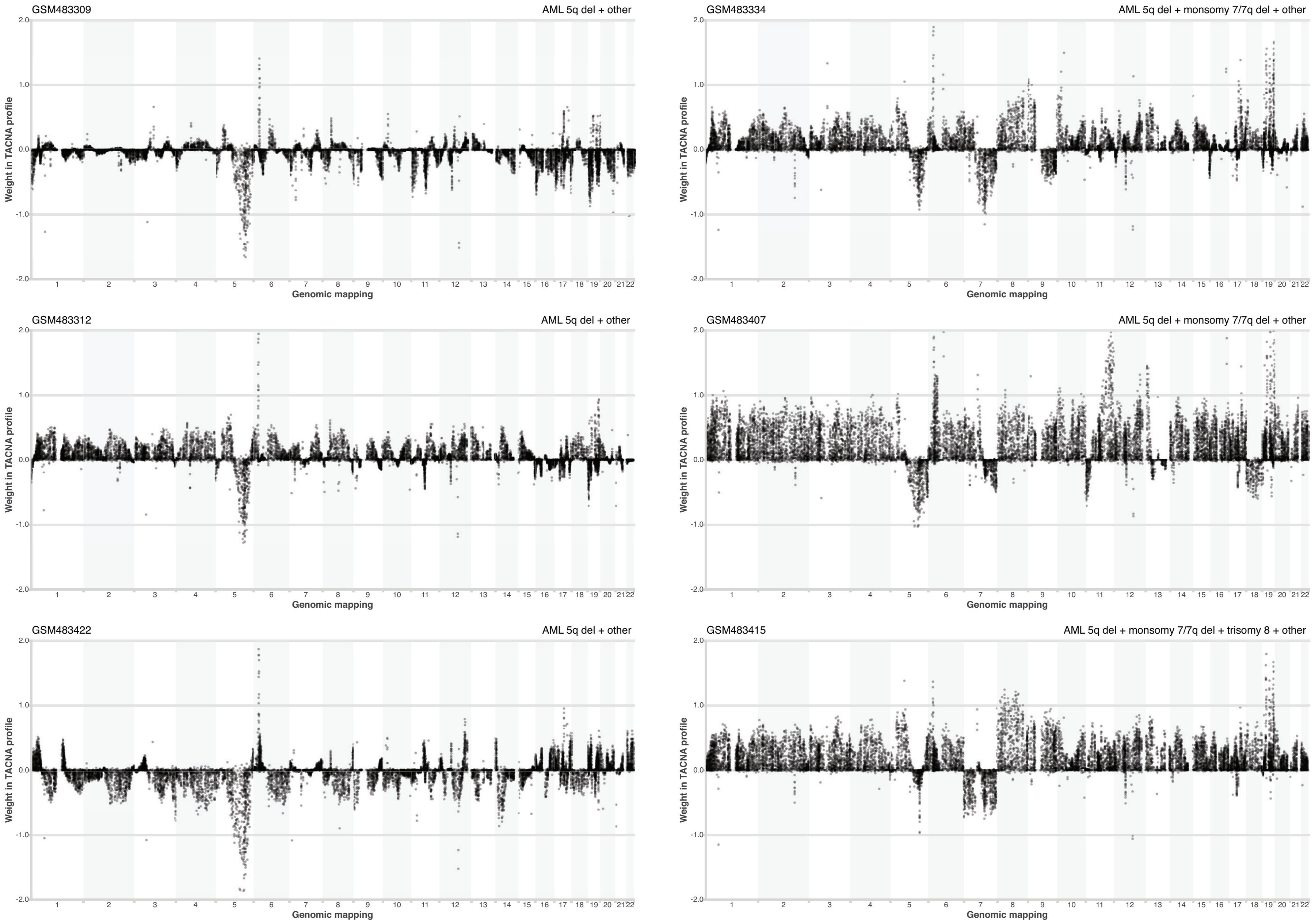

Supplementary Fig. 2 Examples of matching patterns between TACNA profiles and the expected karyotype in acute myeloid leukemia samples in the GEO dataset.

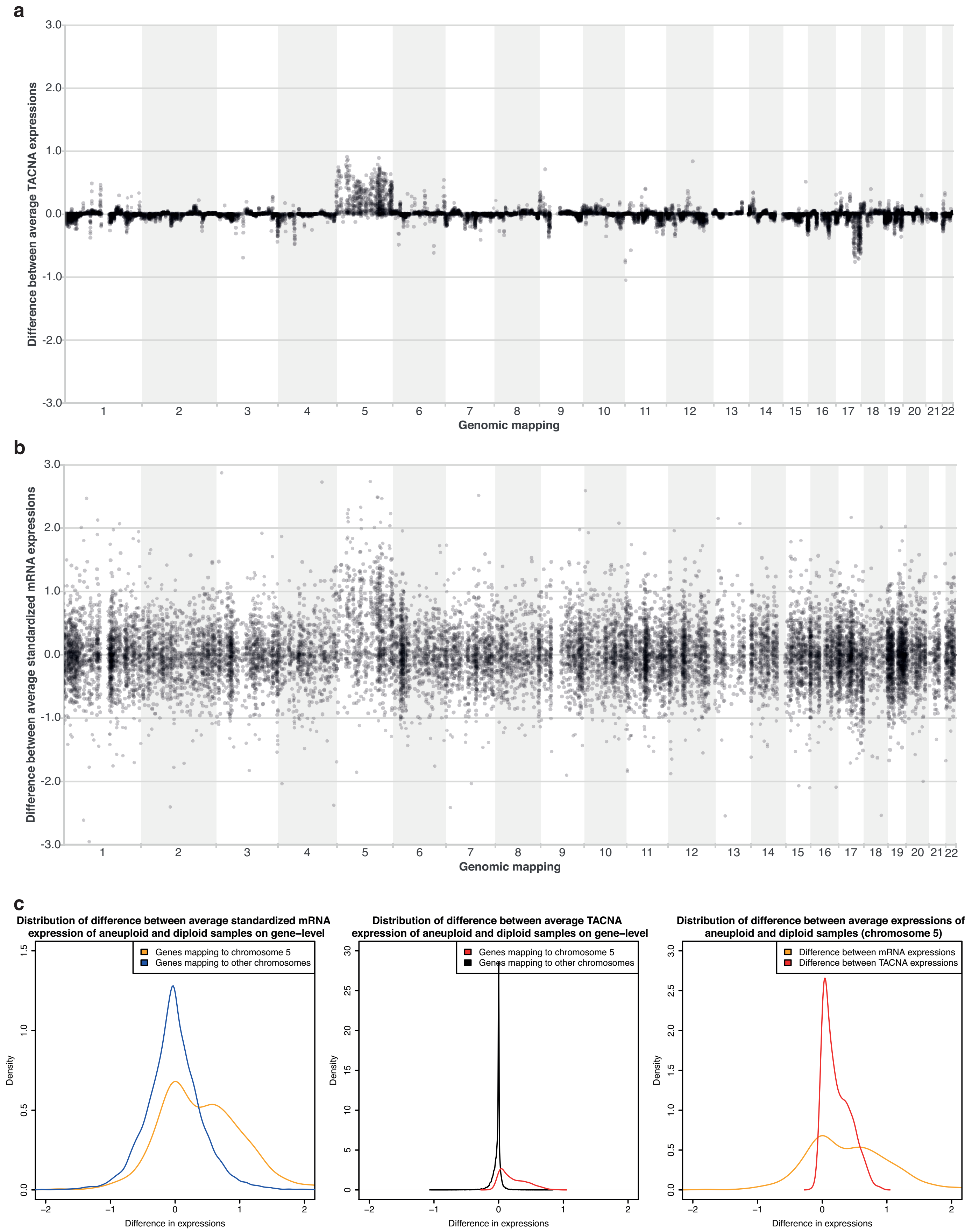

Supplementary Fig. 3 a Differences in expression levels between average TACNA profiles of three aneuploid samples and average TACNA profiles of three diploid samples. **b** Differences in expression levels between average standardized mRNA expression profiles of three aneuploid samples and average standardized mRNA expression profiles of three diploid samples. **c** Left: distribution of the differences in expression levels between average standardized mRNA expression of aneuploid and diploid samples on gene-level separately for chromosome 5 and other chromosomes. Middle: distribution of the differences in expression levels between average TACNA expression of aneuploid and diploid samples on gene-level separately for chromosome 5 and other chromosomes. Right: comparison between average differences in TACNA expression levels and average differences in standardized mRNA expression levels for chromosome 5.

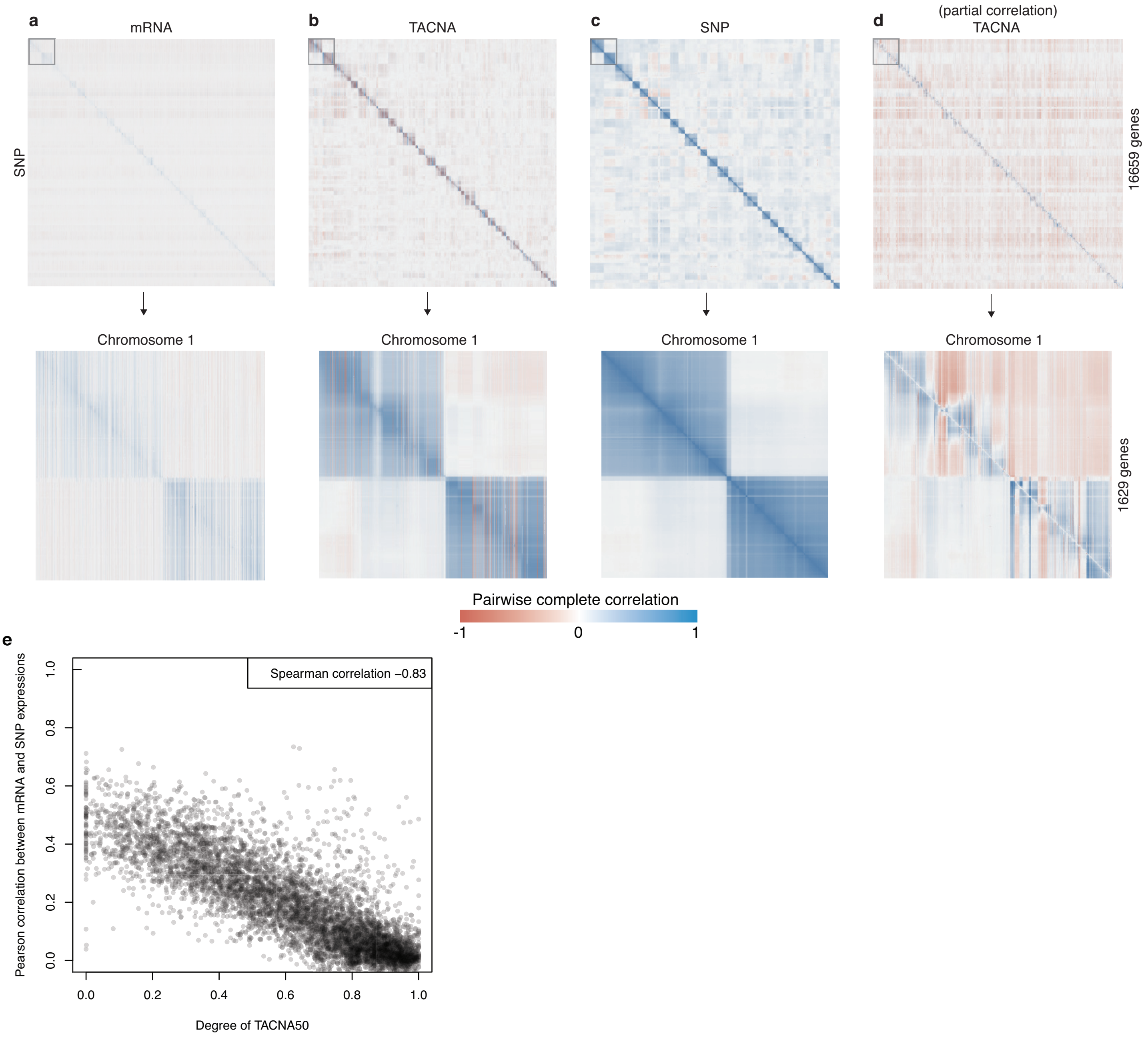

Supplementary Fig. 4 a Correlation between mRNA expression profiles and CNA profiles (derived from SNP) from TCGA dataset on the gene-level. **b** Correlation between TACNA profiles and CNA profiles (derived from SNP) from TCGA dataset on the gene-level. **c** Association within CNA profiles (derived from SNP) from TCGA dataset on the gene-level. **d** Partial correlation coefficients between TACNA profiles and CNA profiles (derived from SNP) from TCGA dataset on the gene-level. Insets of each panel represents correlation coefficients for genes mapping to chromosome 1. **e** Scatter plot of the degree of TACNA versus Pearson correlations between mRNA and SNP expressions for genes occurring once in an extreme-valued region of a CNA-CES in the TCGA dataset. Only CNA-CESs with >50 genes in their extreme-valued region were considered.

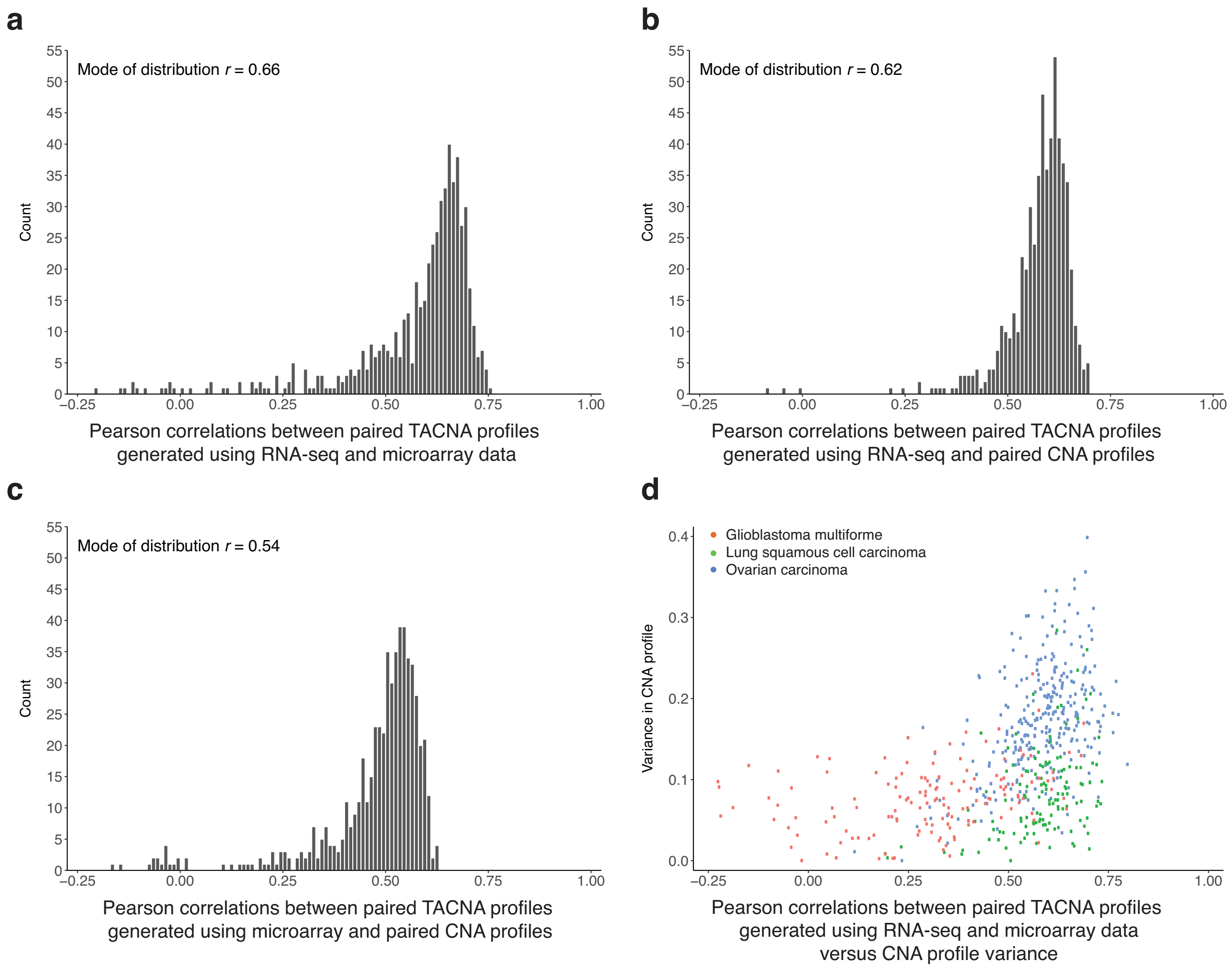

Supplementary Fig. 5. a Pearson correlations between paired TACNA profiles derived from RNA sequencing and microarray profiles (*n* = 570). **b** Distribution of Pearson correlations between TACNA profiles derived from RNA sequencing data and paired CNA profiles. **c** Distribution of Pearson correlations between TACNA profiles derived from microarray data and paired CNA profiles. **d** Variance observed in CNA profiles versus Pearson correlations between TACNA profiles derived from RNA sequencing data and microarray data.

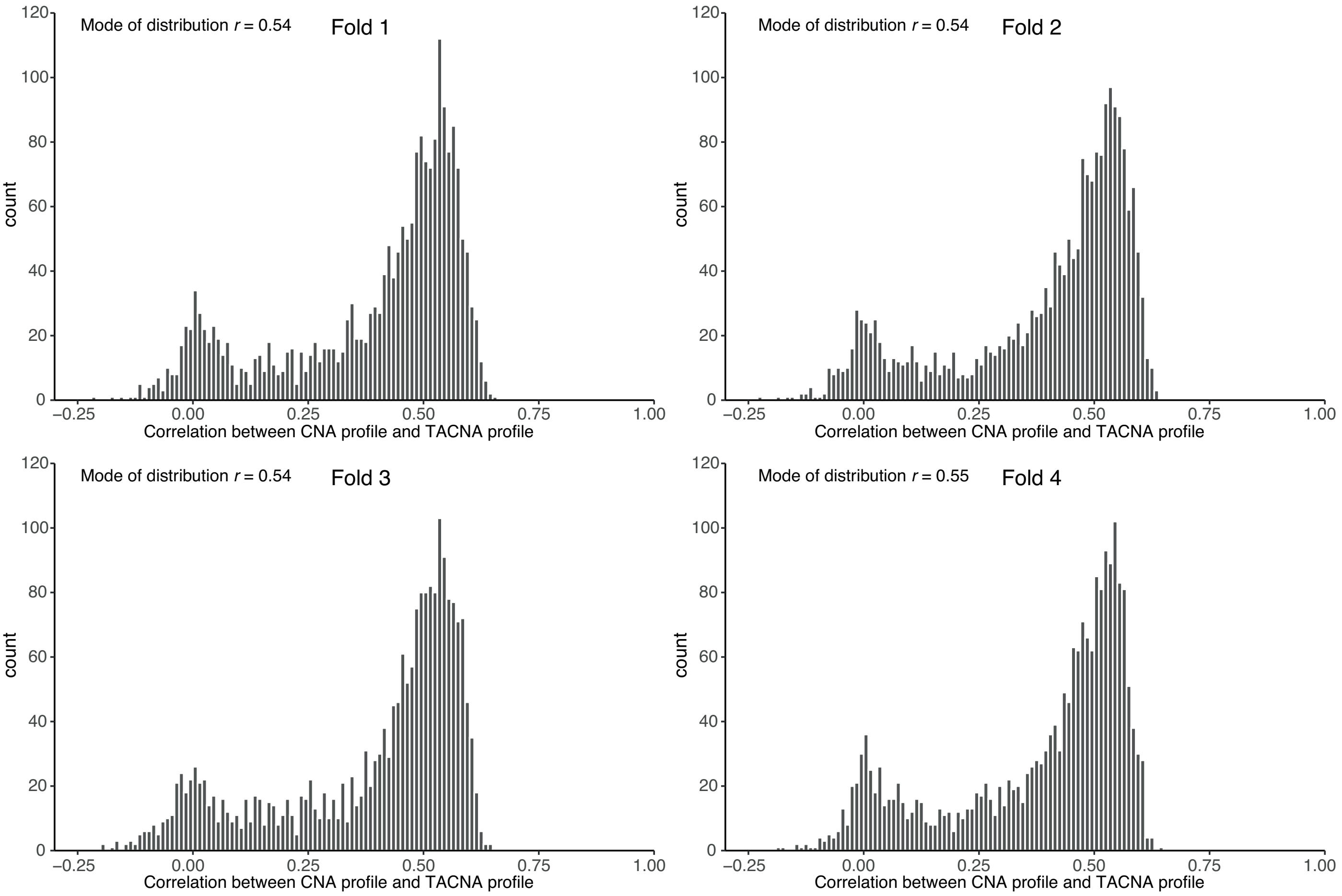

Supplementary Fig. 6 Cross-validation analysis in the TCGA dataset. Distributions of Pearson correlation coefficients between TACNA profiles of randomly chosen 20% of samples of the TCGA dataset (RNA sequencing) in each fold using CNA-CESs derived from the remaining 80% of samples of the TCGA dataset and paired CNA profiles (derived from SNP).

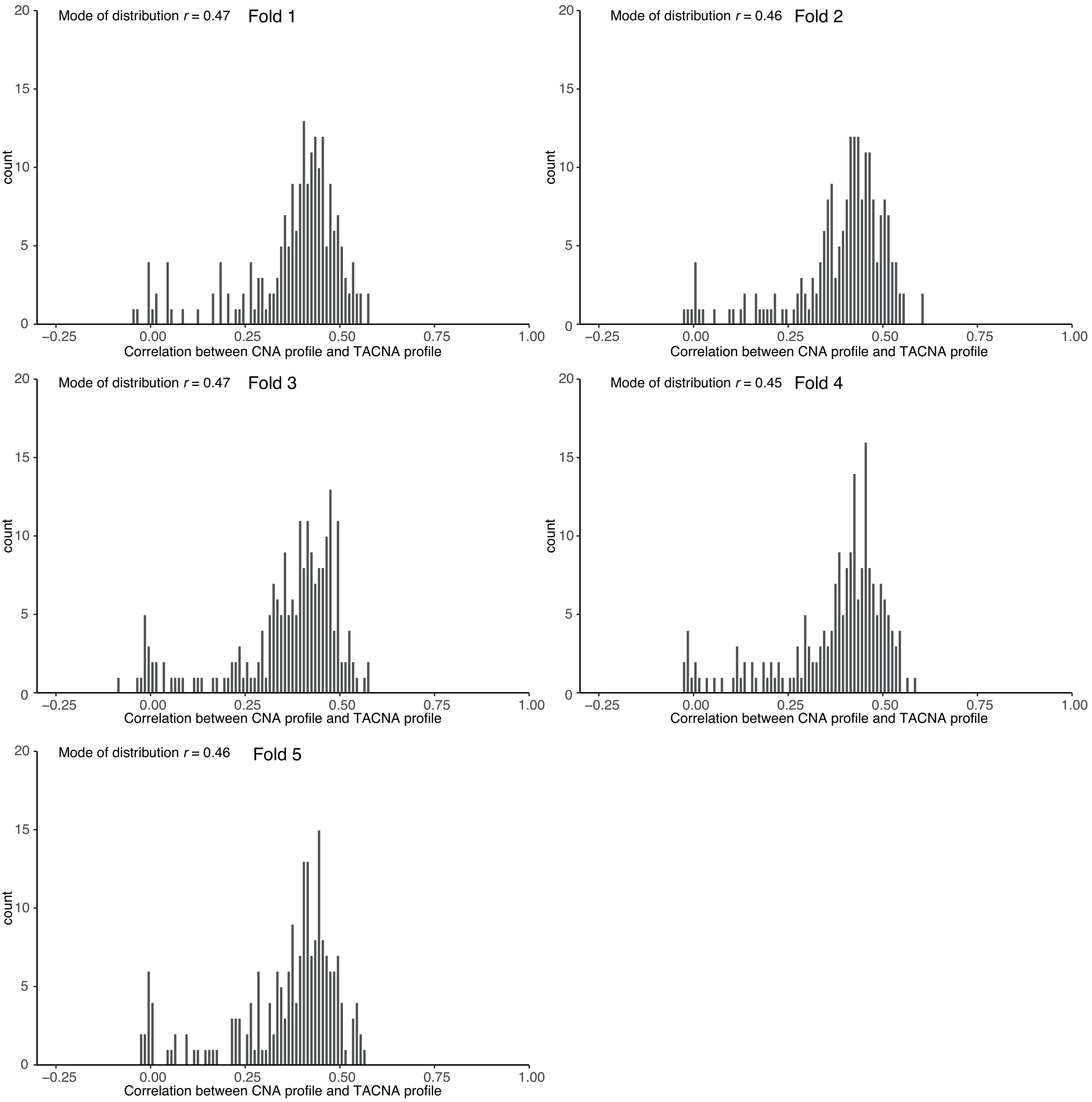

Supplementary Fig. 7 Cross-validation analysis in the CCLE dataset. Distributions of Pearson correlation coefficients between TACNA profiles of randomly chosen 20% of samples of the CCLE dataset (microarray) in each fold using CNA-CESs derived from the remaining 80% of samples of the CCLE dataset and paired CNA profiles (derived from SNP).

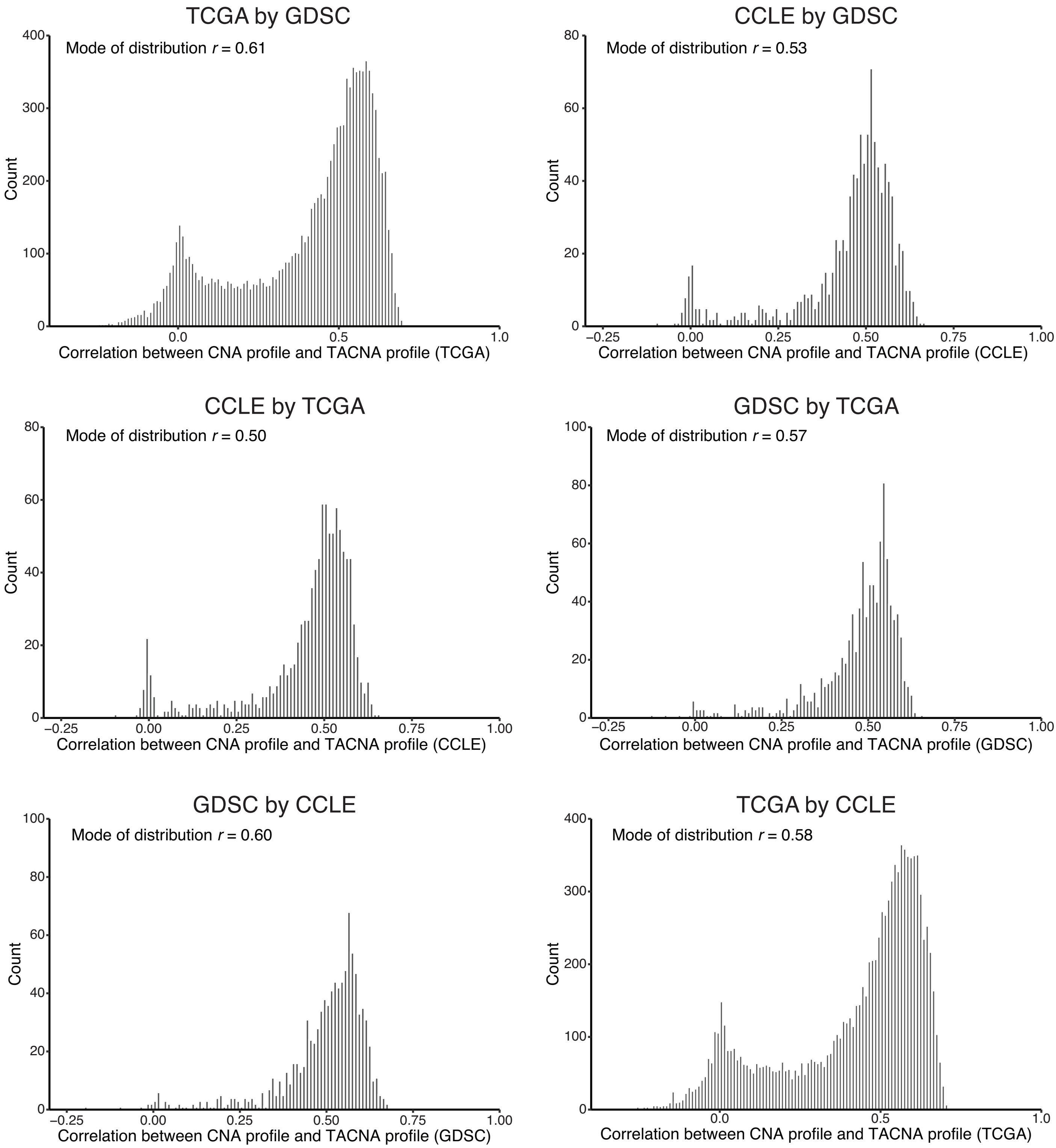

Supplementary Fig. 8 Cross-study cross-validation. Distributions of Pearson correlation coefficients between TACNA profiles of dataset *i* using CNA-CESs derived from dataset *j* and paired CNA profiles (derived from SNP) of dataset *i* (i by *j* where *i* and *j* are from TCGA, CCLE & GDSC).

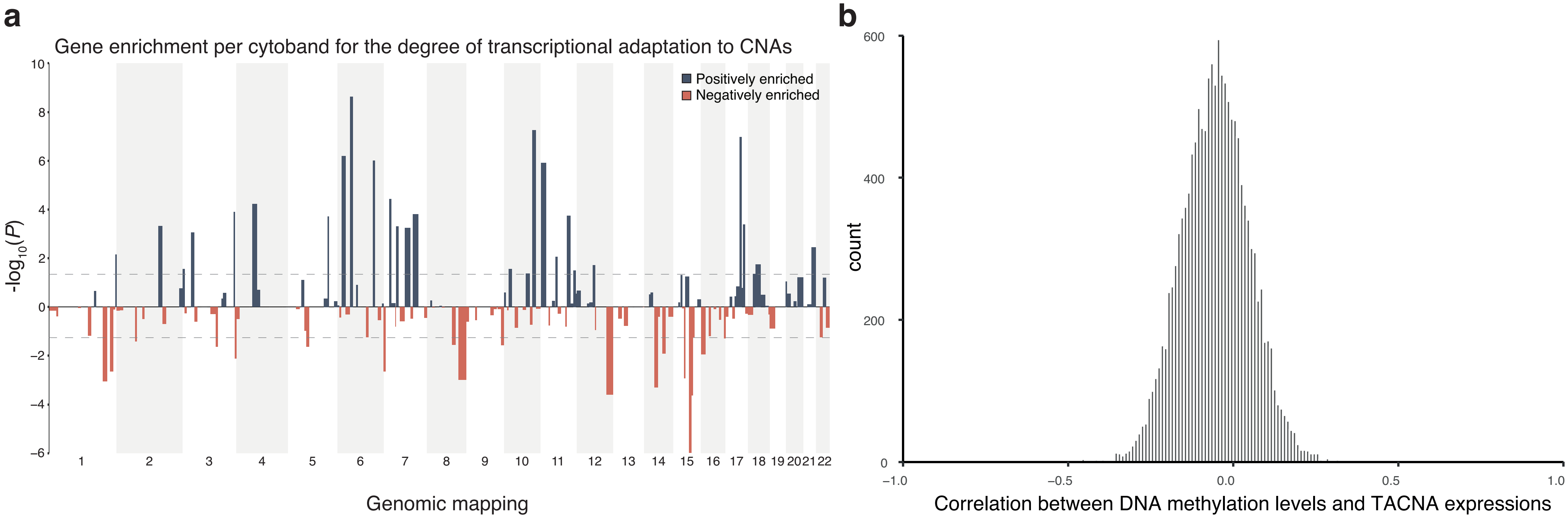

Supplementary Fig. 9 a Manhattan plot showing the -log10(P value) enrichment value on the y-axis for the degree of transcriptional adaptation of genes per cytogenetic band defined according to the MSigDB Positional Gene Sets collection. The average degree of transcriptional adaptation was calculated for genes occurring once in a CNA-CES in both the GEO and TCGA dataset. The dotted lines represent a significance level of P = 0.05. **b** Spearman correlation between the mean methylation levels of individual genes and their degree of transcriptional adaptation in a subset of samples (n = 9,317) from the TCGA dataset.

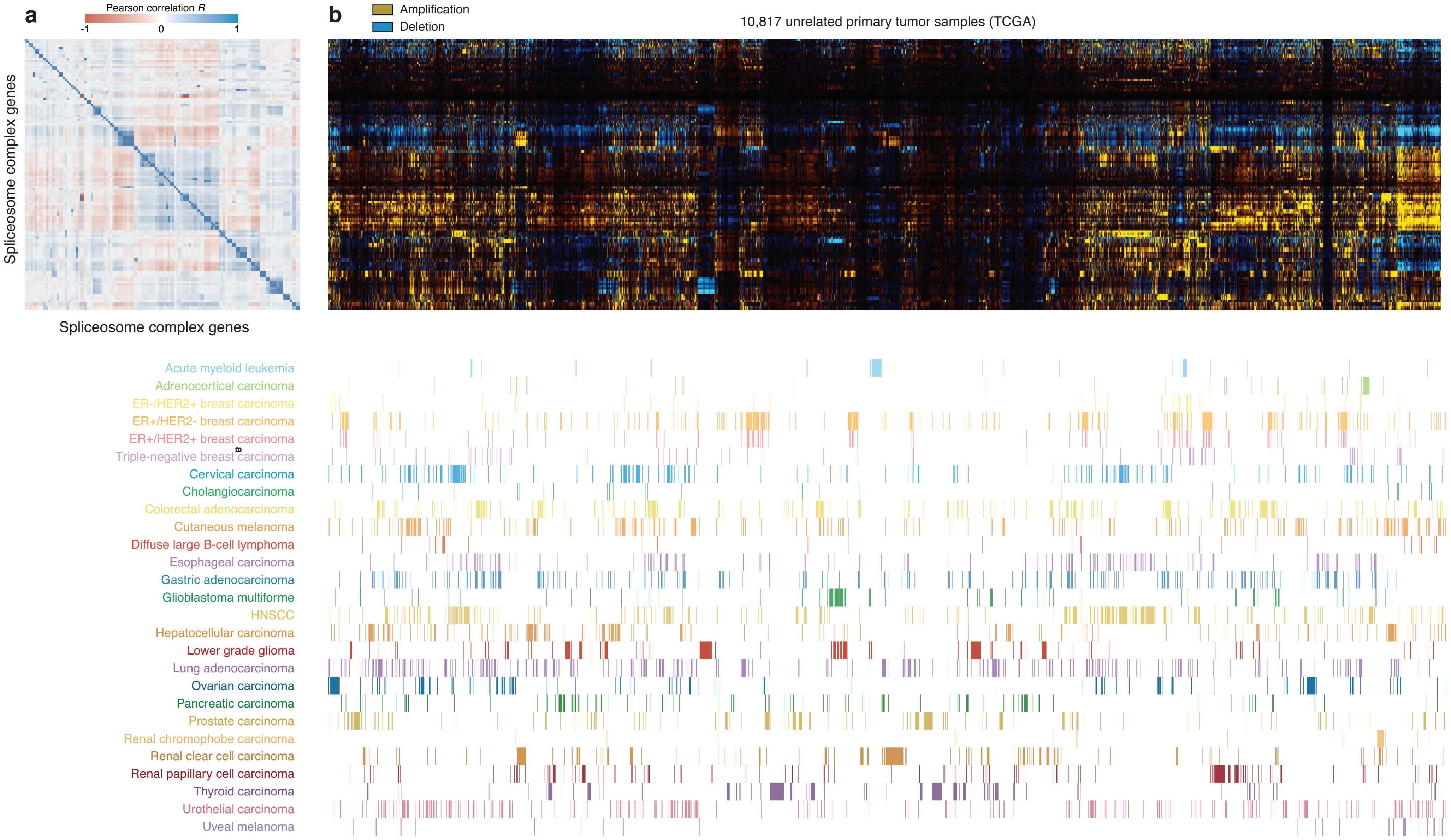

Supplementary Fig. 10 a Hierarchical clustering of the correlation distance matrix of genes belonging to the Spliceosome complex (CORUM) in the TCGA-dataset. **b** Hierarchical clustering of the transcriptional effects of CNAs in the TCGA dataset for genes belonging to the Spliceosome complex (CORUM) for 10,817 samples.

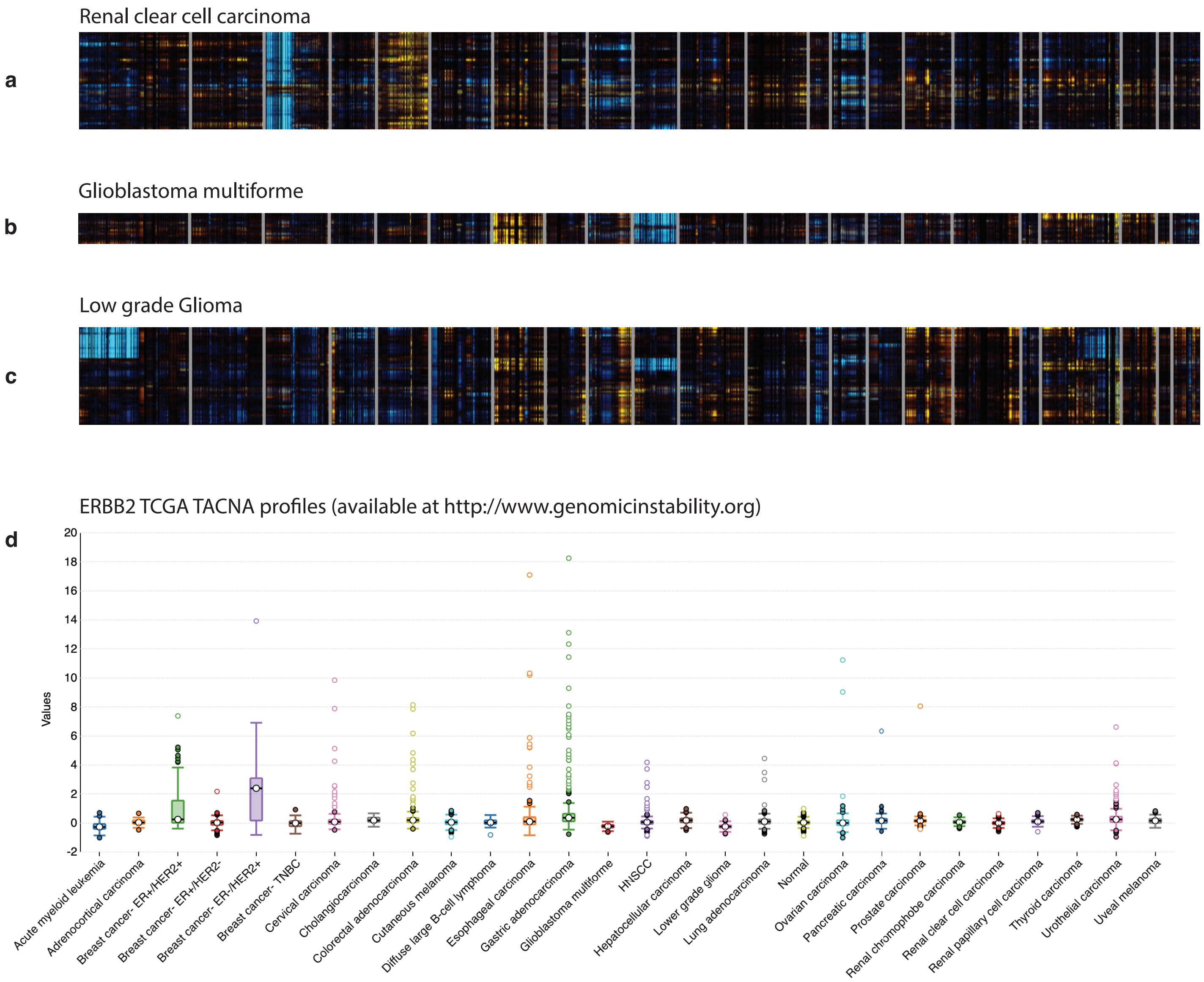

Supplementary Fig. 11 a TACNA profiles for renal clear cell carcinoma in the TCGA dataset. **b** TACNA profiles for glioblastoma multiforme in the TCGA dataset. **c** TACNA profiles for low-grade glioma in the TCGA dataset. **d** Example of exploring *ERBB2* TACNA values across samples in the TCGA dataset at www. genomicinstability.org. The bars represent the interquartile range.The centre line of each bar represents median. The whiskers are defined as 1.5 x interquartile range. Adrenocortical carcinoma (n = 79), Colorectal adenocarcinoma (n = 573), Ovarian carcinoma (n = 307), ER+/HER2+ breast carcinoma (n = 140), ER+/HER2- breast carcinoma (n = 554), ER-/HER2+ breast carcinoma (n = 43), Glioblastoma multiforme (n = 166), Renal papillary cell carcinoma (n = 291), Cholangiocarcinoma (n = 36), Hepatocellular carcinoma (n = 373), Cutaneous melanoma (n = 472), Gastric adenocarcinoma (n = 415), Esophageal carcinoma (n = 185), Lung adenocarcinoma (n = 517), Uveal melanoma (n = 80), Diffuse large B−cell lymphoma (n = 48), Cervical carcinoma (n = 306), Prostate carcinoma (n = 498), Renal clear cell carcinoma (n = 534), Renal chromophobe carcinoma (n = 66), Urothelial carcinoma (n = 408), Thyroid carcinoma (n = 509), Pancreatic carcinoma (n = 179), HNSCC (n = 522), Lower grade glioma (n = 530), Acute myeloid leukemia (n = 173), Triple-negative breast carcinoma (n = 146).

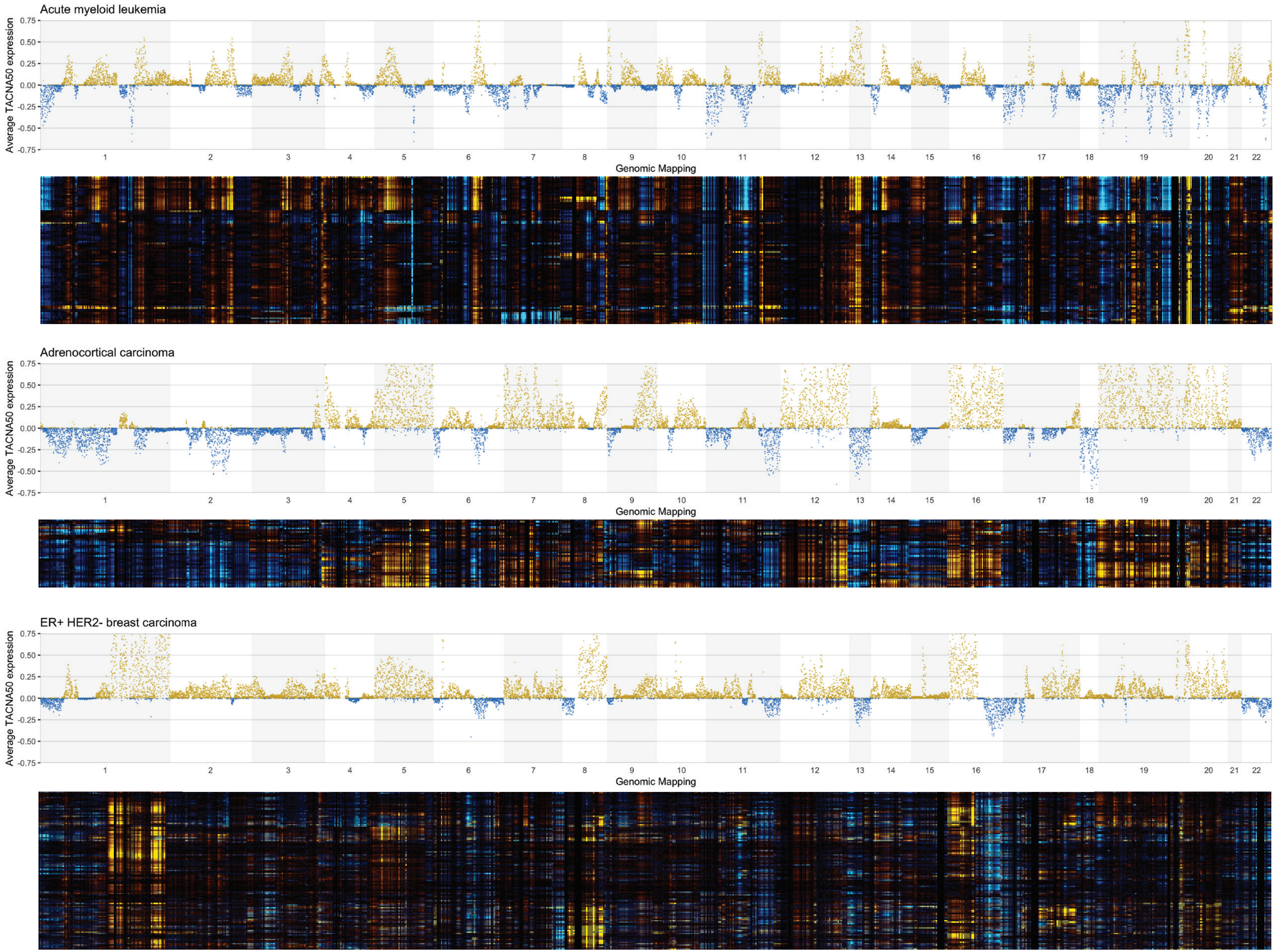

Supplementary Fig. 12 Heatmap of TACNA profiles and average TACNA profiles for acute myeloid leukemia, adrenocortical carcinoma and ER-positive/HER2-negative breast carcinoma in the TCGA dataset.

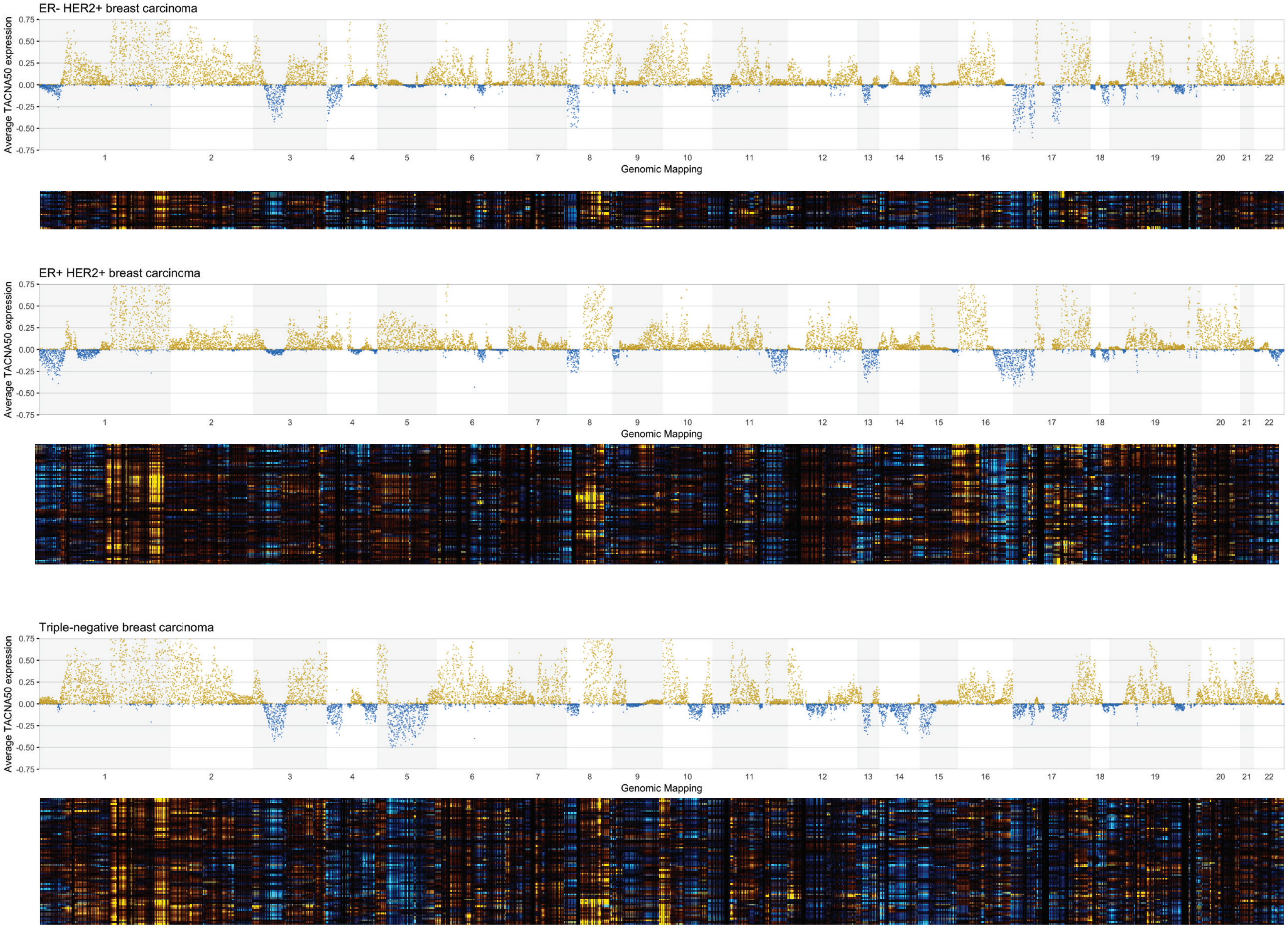

Supplementary Fig. 13 Heatmap of TACNA profiles and average TACNA profiles for ER-negative/HER2-positive breast carcinoma, ER-positive/HER2-positive breast carcinoma and triple-negative breast carcinoma in the TCGA dataset.

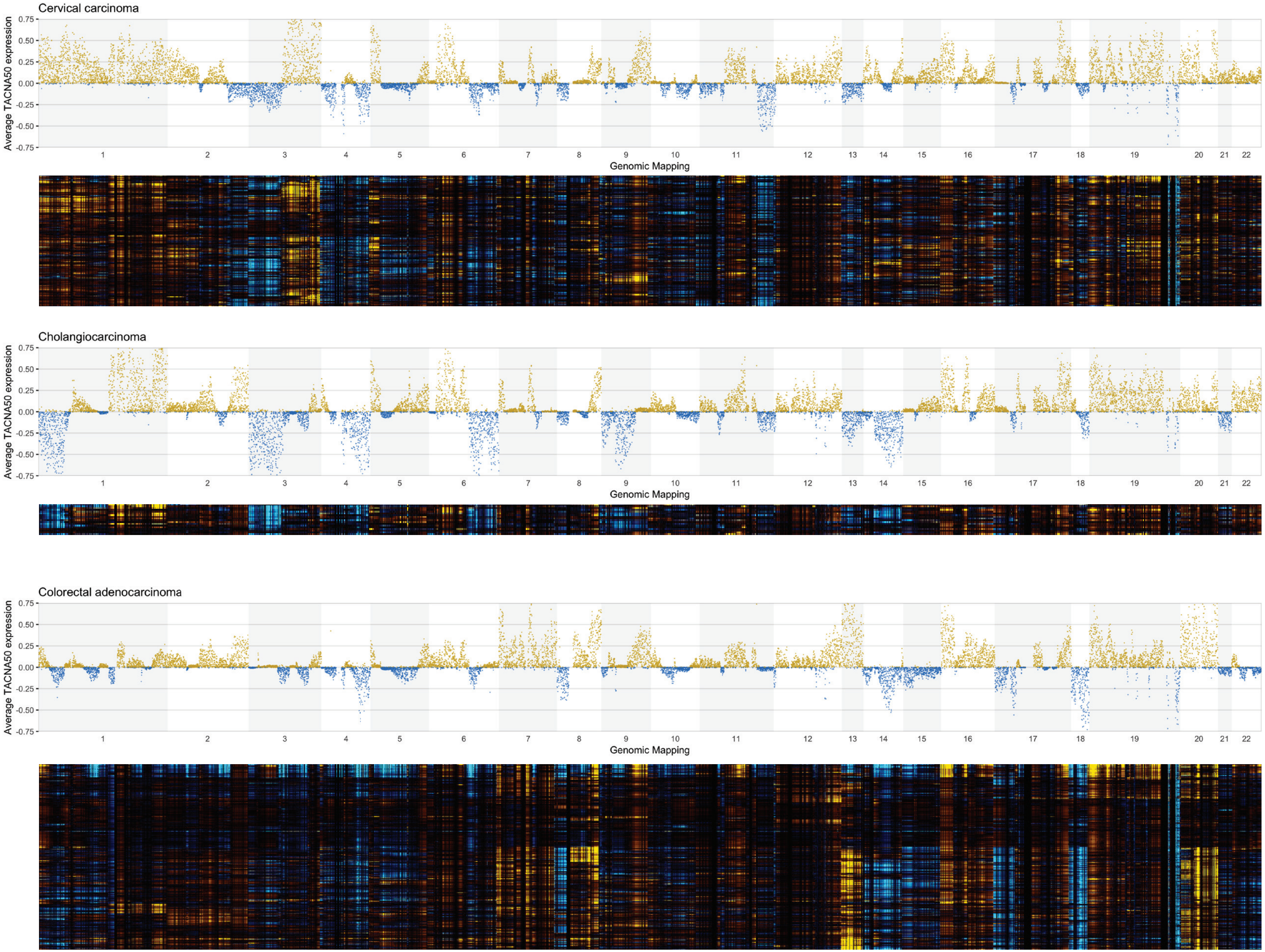

Supplementary Fig. 14 Heatmap of TACNA profiles and average TACNA profiles for cervical carcinoma, cholangiocarcinoma and colorectal adenocarcinoma in the TCGA dataset.

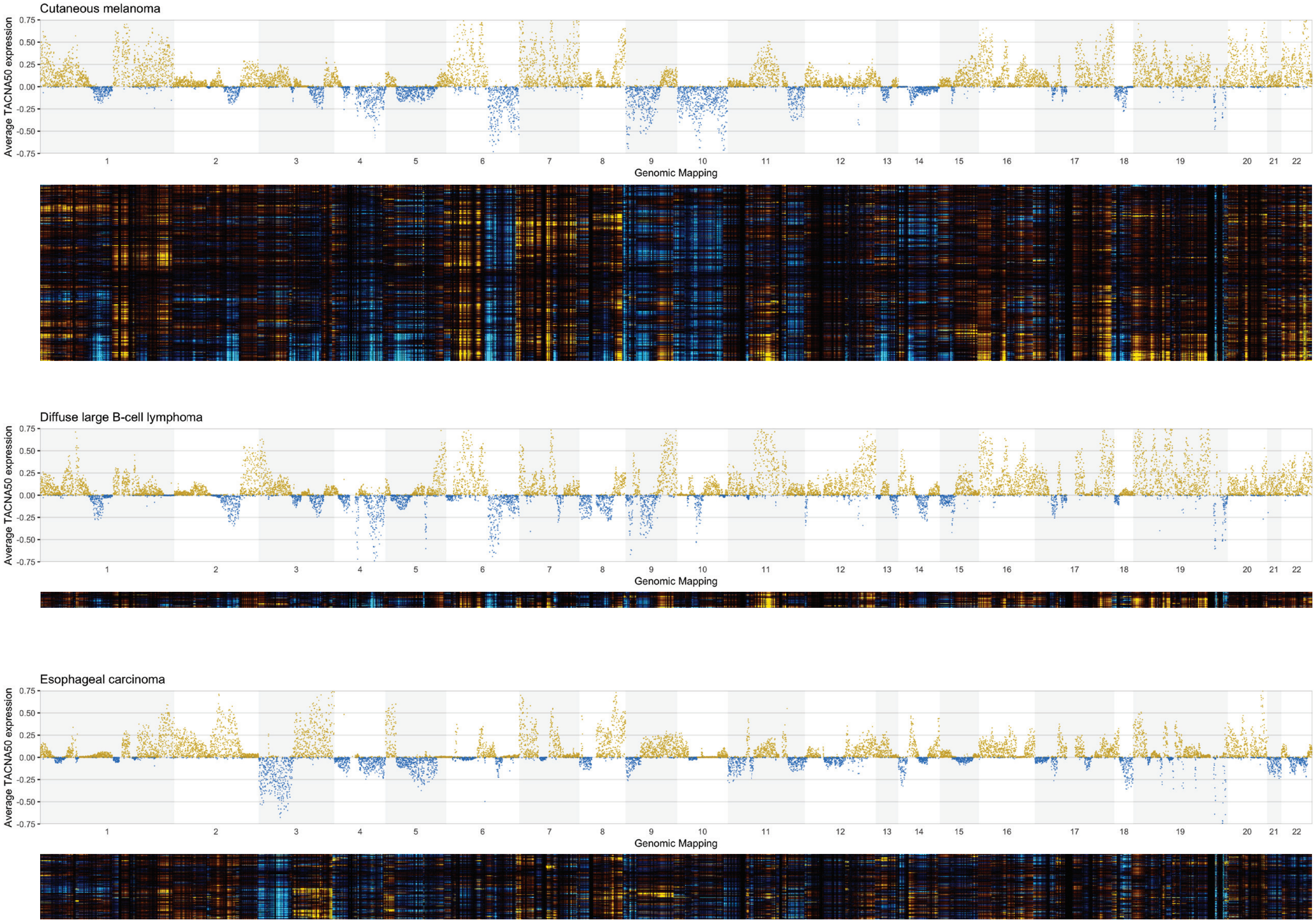

Supplementary Fig. 15 Heatmap of TACNA profiles and average TACNA profiles for cutaneous melanoma, diffuse large B-cell lymphoma and esophageal carcinoma in the TCGA dataset.

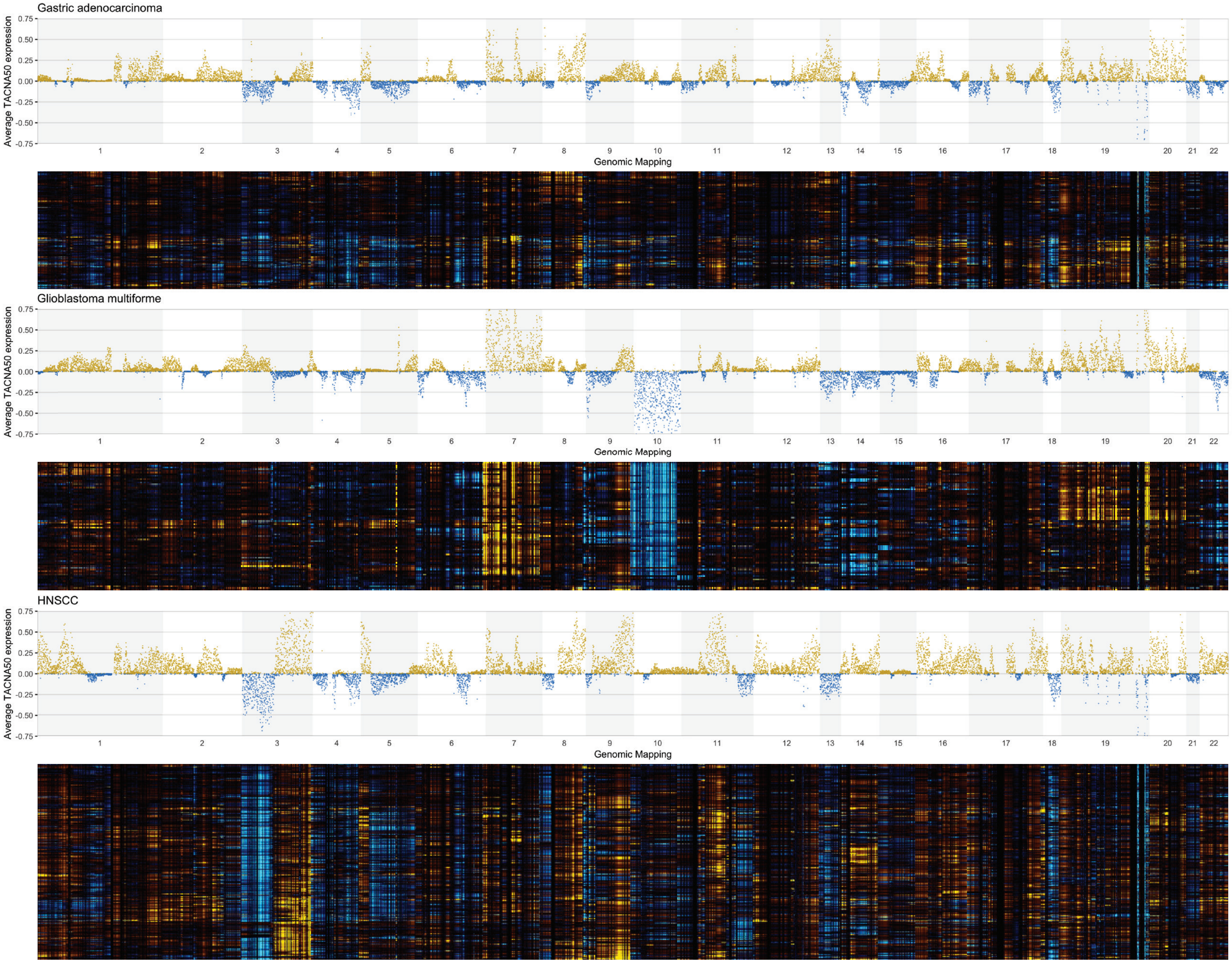

Supplementary Fig. 16 Heatmap of TACNA profiles and average TACNA profiles for gastric adenocarcinoma, glioblastoma multiforme and head and neck squamous cell carcinoma (HNSCC) in the TCGA dataset.

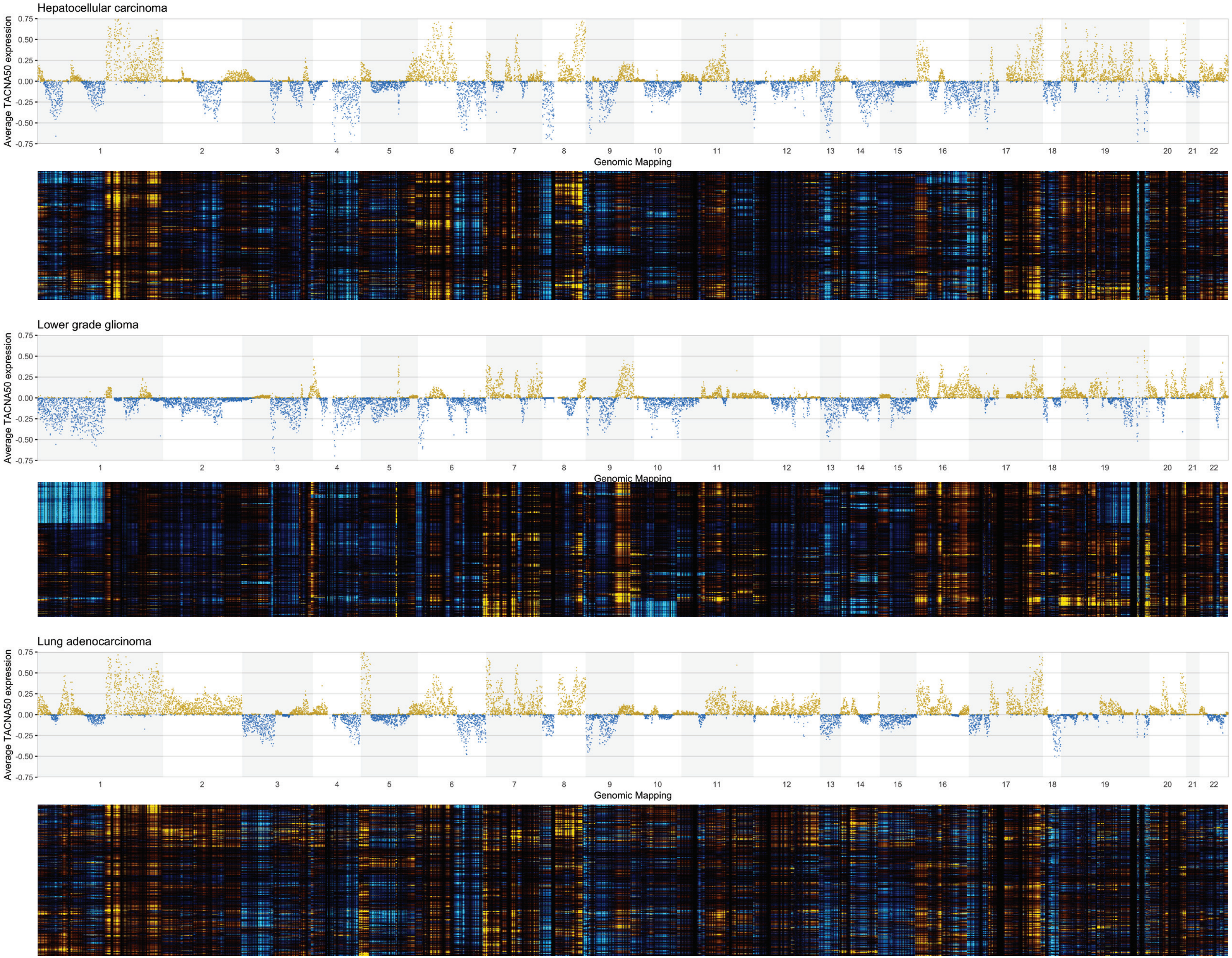

Supplementary Fig. 17 Heatmap of TACNA profiles and average TACNA profiles for hepatocellular carcinoma, lower grade glioma and lung adenocarcinoma in the TCGA dataset.

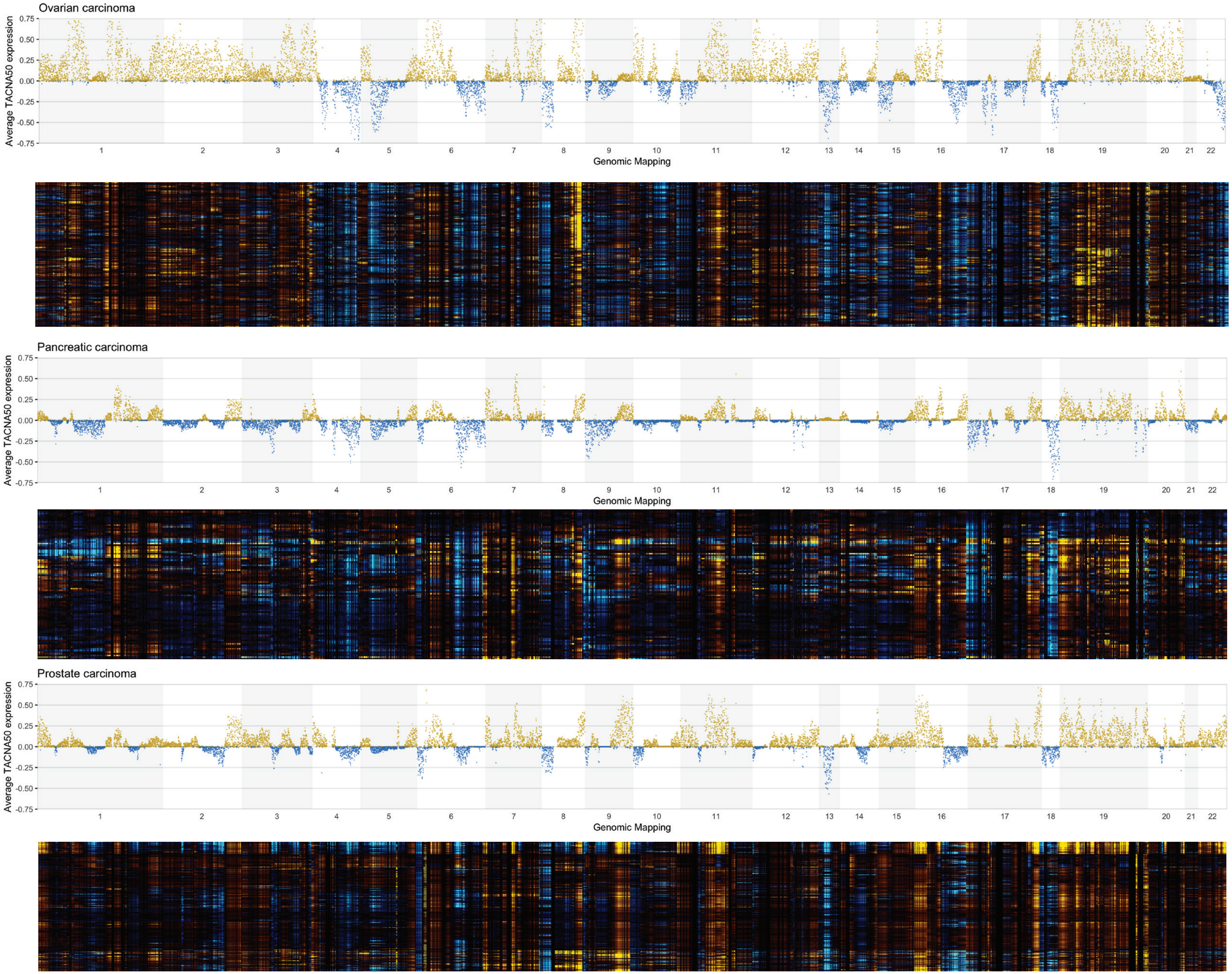

Supplementary Fig. 18 Heatmap of TACNA profiles and average TACNA profiles for ovarian carcinoma, pancreatic carcinoma and prostate carcinoma in the TCGA dataset.

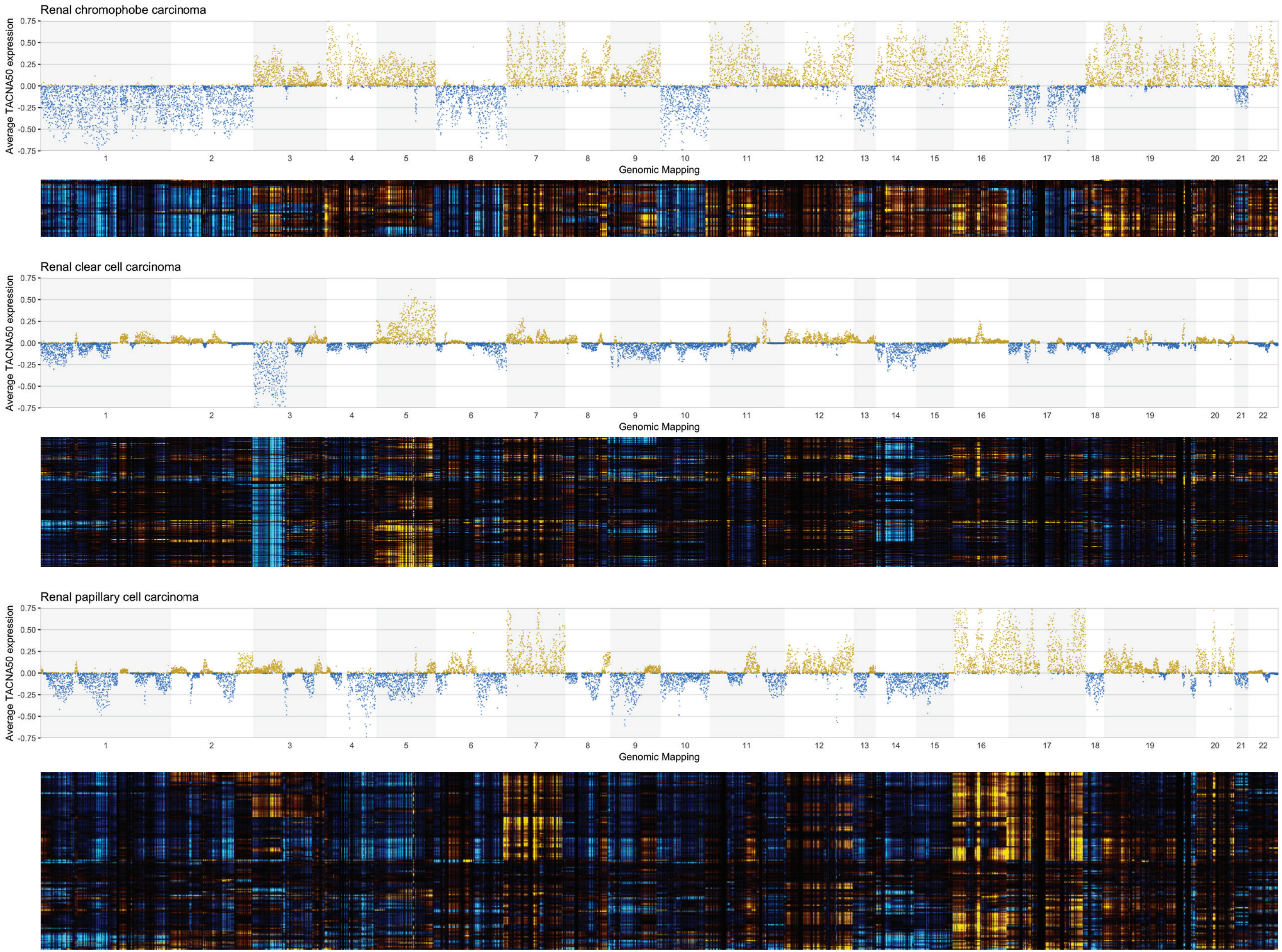

Supplementary Fig. 19 Heatmap of TACNA profiles and average TACNA profiles for renal chromophobe carcinoma, renal clear cell carcinoma and renal papillary cell carcinoma in the TCGA dataset.

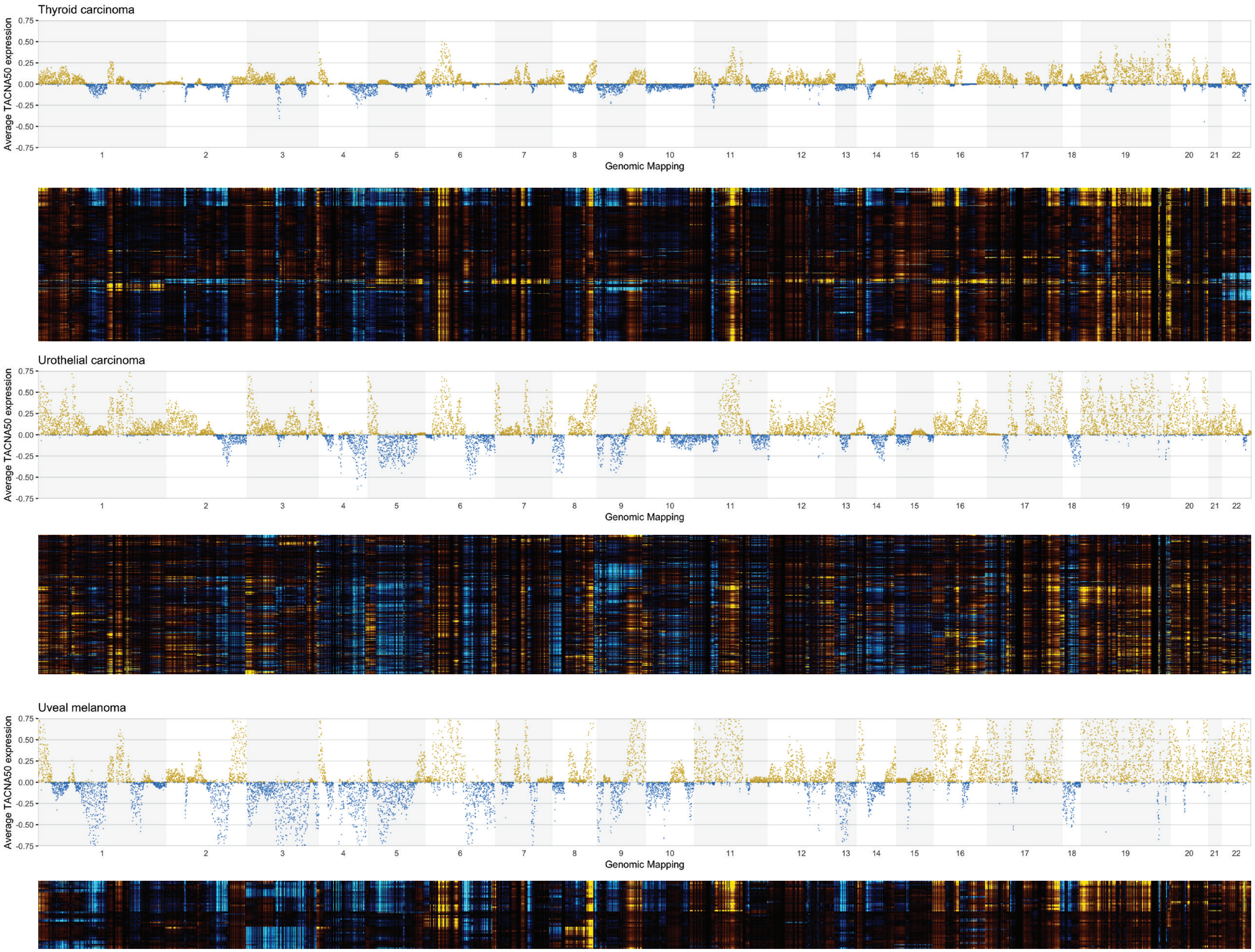

Supplementary Fig. 20 Heatmap of TACNA profiles and average TACNA profiles for thyroid carcinoma, urothelial carcinoma and uveal melanoma in the TCGA dataset.

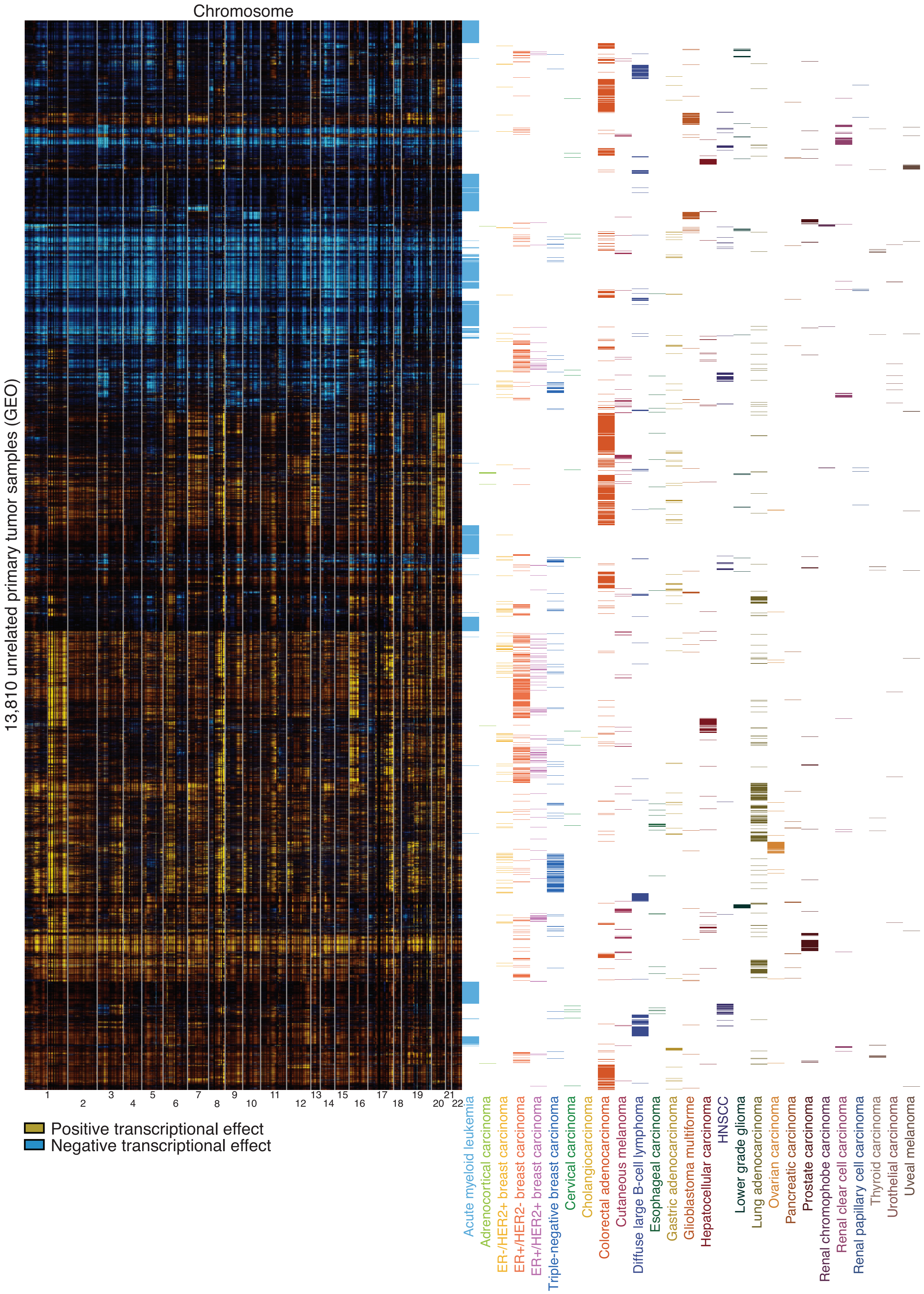

Supplementary Fig. 21 Hierarchical clustering of the landscape of transcriptional effects of CNAs in the GEO dataset for 13,810 samples from tumor types also present in the TCGA dataset.

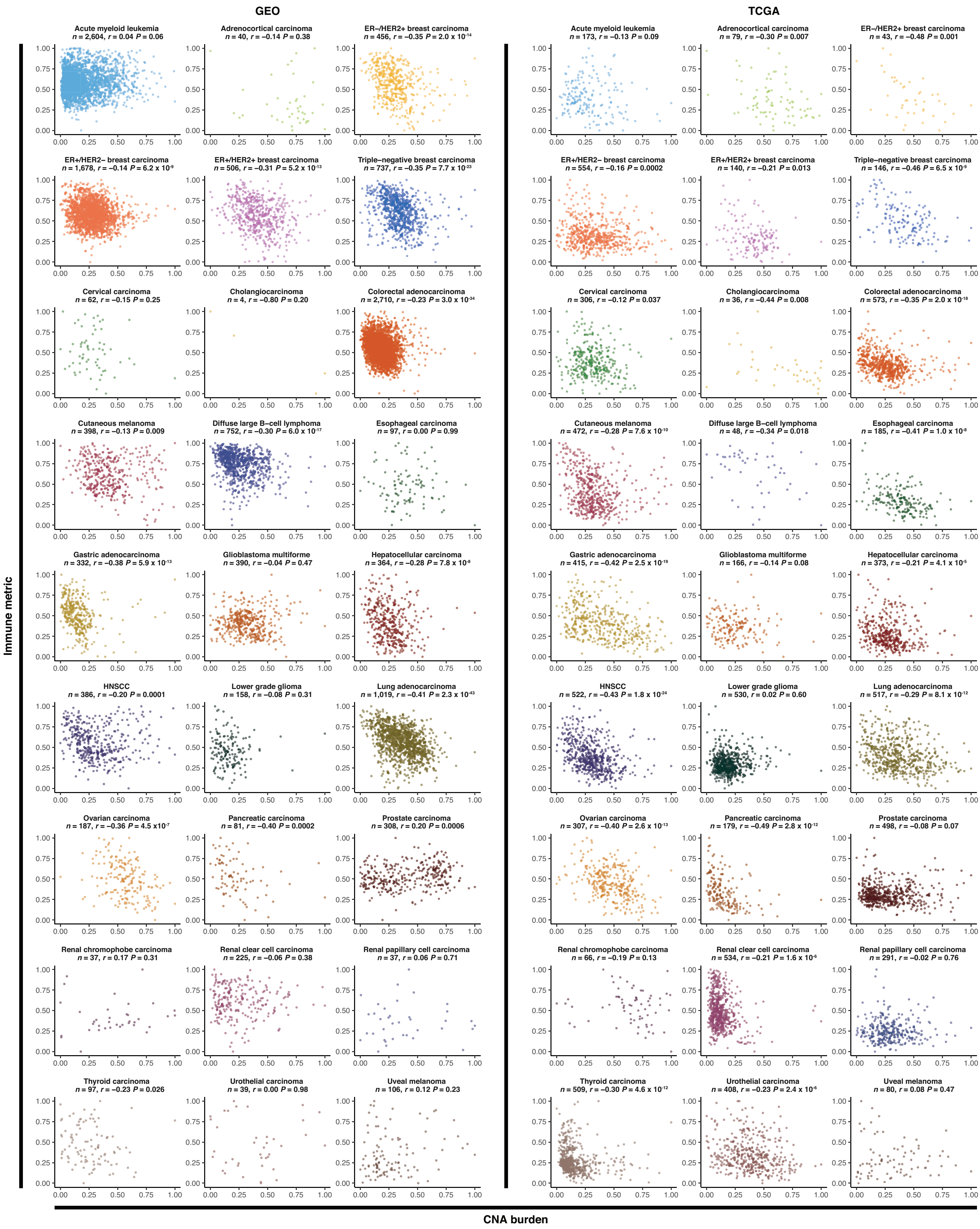

Supplementary Fig. 22 Per tumor type, Spearman correlations between inferred CNA burden and an expression-based metric describing CD8+ T cell activity for each sample in the GEO and TCGA datasets. Both inferred CNA burden and metrics were normalized per tumor type.

Supplementary Note 1

Data acquisition

GEO dataset

Publicly available microarray expression data generated with Affymetrix HG-U133 Plus 2.0 was obtained from GEO (accession number GPL570).[1] To select healthy or cancer tissue samples, we applied a two-step search strategy – automatic filtering on keywords followed by manual curation. This search strategy was applied to the Simple Omnibus Format in Text (SOFT) files obtained from GEO for GPL570, which contains metadata for each individual sample, including experimental conditions and patient information. In the automatic filtering step, samples were retained if one of the keywords could be matched in any of the associated descriptive fields in the SOFT file. The keywords used in the automatic filtering were chosen in such a way that the chance to miss relevant samples would be minimized (e.g. colon, breast, lung). Because this automatic filtering was aimed at sensitivity, not specificity, a manual curation step was necessary to obtain a list containing only relevant samples. In the manual curation step, samples were retained if they represented healthy or cancer tissue obtained from patients and raw data (CEL files) were available. Samples from cell lines, cultured human biopsies and animal-derived tissue were excluded. This dataset is referred to as the GEO dataset throughout this manuscript.

TCGA dataset

From TCGA, we obtained the pre-processed and normalized level 3 RNA-seq (version 2) data for 34 cancer datasets available at the Broad GDAC Firehose portal (downloaded January 2017

https://gdac.broadinstitute.org/). For each sample, we downloaded RNA-Seq with Expectation Maximization (RSEM) gene normalized data (identifier: illuminahiseq_rnaseqv2-RSEM_genes_normalized).[2] RNA-Seq expression level read counts were normalized using FPKM-UQ (Fragments per Kilo-base of transcript per Million mapped reads upper quartile normalization).[3] This dataset is referred to as the TCGA dataset throughout this manuscript. In addition, we collected pre-processed segmented somatic CNA data for each of the 34 cancer datasets (identifier: genome_wide_snp_6-segmented_scna_minus_germline_cnv_hg19), which was generated with the Affymetrix Genome-Wide Human SNP Array 6.0. In short, the copy number segmentation pipeline implemented by TCGA and applied to Affymetrix SNP Array 6.0 uses a fully open-source tool Birdsuite and the DNAcopy R-package to perform a circular binary segmentation (CBS) analysis.[4,5] CBS translates noisy intensity measurements into chromosomal regions of equal copy number. The final output files are segmented into genomic regions with the estimated copy number for each region. Next, the copy number values are transformed into segment mean values, which are equal to $\log_2$(copy number/2).[3]

CCLE dataset

Raw gene expression data was obtained from the CCLE project (downloaded February 2017), which conducted a detailed genetic characterization of a large panel of human cancer cell lines.[6] Expression data within the CCLE project was generated with Affymetrix HG-U133 Plus 2.0. This dataset is referred to as the CCLE dataset throughout this manuscript. In addition, pre-processed somatic CNA data was obtained containing segmented normalized $\log_2$ copy number ratios at the gene level. The CNA data was generated with Affymetrix Genome-Wide Human SNP Array 6.0.[6]

In short, CBS was applied to segment the normalized copy number estimates followed by median centering per sample. The amount of copy numbers for each gene was defined as the maximum absolute segmented copy number between the start and end base pair of that corresponding gene.

GDSC dataset

From the GDSC portal, we obtained raw expression data generated with Affymetrix HG-U219 (downloaded February 2017).[7] The aim of the GDSC project is to identify molecular features of cancer that predict response to anti-cancer drugs. This dataset is referred to as the GDSC dataset throughout this manuscript. In addition, we obtained the processed segmented somatic CNA data at the individual gene level. This data was generated with Affymetrix Genome-Wide Human SNP Array 6.0 and subsequently processed with the two-stage procedure 'Predict integral copy numbers in cancer' (PICNIC). In the first stage of PICNIC, raw Affymetrix genome-wide SNP 6.0 data are converted into copy number and genotype intensities using previously known genotype structures in normal tissues. Next, a Bayesian Hidden Markov model is applied on this pre-processed data to identify segments of fixed integer allelic copy numbers.[8]

Preprocessing, normalization and quality control

Preprocessing and aggregation of raw expression data within the GEO dataset, CCLE dataset and GDSC dataset was performed according to the robust multi-array average algorithm with RMAExpress (version 1.1.0) using the corresponding latest CDF files provided by Affymetrix.[9] Quality control was performed on the GEO dataset, CCLE dataset and GDSC dataset separately.

For quality control, principal component analysis (PCA) was applied on the sample Pearson product-moment correlation matrix. The first principal component (PCqc) of such an expression microarray correlation matrix describes nearly always a constant pattern that dominates the data, explaining around 80-90% of the total variance. This pattern can be regarded as probe-specific or platform-specific variance, independent of the biological sample hybridized to the array. The correlation of each individual microarray expression profile with this PCqc (in PCA analysis called factor loadings) can be used to detect outliers, as arrays of lesser quality will have a lower correlation with the PCqc. We removed samples with a Pearson $r < 0.8$. Because individual samples could be uploaded multiple times to repositories, we checked our datasets for duplicates. Duplicate CEL files were removed by generating a message-digest algorithm 5 (MD5) hash for each individual CEL file. A MD5 hash acts like a unique fingerprint for each individual file and duplicate CEL files will have an identical MD5 hash. Standardization makes expression values of probesets or genes comparable to each other by removing differences in experiments or platforms to obtain these expression datasets. The expression levels for each probeset (in the GEO dataset, CCLE dataset and GDSC dataset) or gene (in the TCGA dataset) were standardized to a mean of zero and variance of one to remove probeset-specific or gene-specific variability in the datasets.

Independent component analysis

In the present study, ICA was utilized to identify a regulatory model for the mRNA transcriptome.[10] One can envision two extreme versions for the regulatory model of the mRNA transcriptome. On the one extreme side, a model can be defined where each individual gene has

its own regulatory factor. On the other extreme side, all genes are regulated by just one regulatory factor. Applying ICA on a large mRNA expression dataset with p genes and n samples allows one to gain insight into the dimensionality of the mRNA transcriptome regulation. In addition, this model will give insight into how a specific regulatory factor influences the mRNA expression levels of individual genes.

In ICA, a pre-processing technique called whitening is applied on the input dataset to make the estimation more time efficient. In the present study, whitening was used to transform the input dataset ($X_{p\times n}$) containing mRNA expression profiles of p probesets or genes from n samples linearly into a new dataset ($X'_{p\times n}$) where all *n* transformed samples are orthogonal (i.e. uncorrelated and their variance equals one). In other words, whitening transformed $X_{p\times n}$ in such a way that the covariance matrix between transformed samples became $I_{n\times n}$ (identity matrix).

Next, ICA was conducted on the whitened mRNA expression dataset ($X'_{p\times n}$) resulting into extraction of i independent components (each of dimension $p \times 1$ and $i \leq n$) and a mixing matrix (MM) of dimension $i \times n$. To choose i, PCA was conducted on the covariance matrix between samples of $X'_{p\times n}$. After that, i was chosen as the number of top principal components which captured 85% of the total variance seen in $X'_{p\times n}$. The individual independent components resulting from ICA are referred to as estimated sources ($\mathrm{ES}_{p\times 1}$) and the matrix with all the ESs in different columns is referred to as the estimated source matrix ($\mathrm{ESM}_{p\times i}$). Each ES is statistically independent of any other ES. In other words, information about scalars of one ES does not give any information about scalars of any other ES. Also, each ES captures a different part of variation observed in the mRNA expression data and scalars of an ES are not correlated with scalars of any other ES. We hypothesized that each ES captures the effects of an underlying regulating factor

on gene expression levels. In the MM each column corresponds to an ES and each row corresponds to a sample. Each column of the MM contains the coefficients for samples which can be seen as an indirect measurement of 'activity' of the underlying regulatory factor in the sample under investigation. Each weight in an ES represents how strong the underlying regulatory factor influences the expression level of the corresponding probeset or gene. The sign of a weight in an ES defines the direction of the change in mRNA expression in relation to the 'activity' of the underlying regulatory factor in the sample under investigation. The inner product between the vector of 'coefficients' of an individual sample in the MM and the vector of ES weights per individual probeset or gene results in the original mRNA expression level. That is, the following equation holds:

$$X'_{p\times n} = \left(\mathrm{ESM}_{p\times i}\right) \times (\mathrm{MM}_{i\times n})$$

$$\Longrightarrow (X'_{p\times n}) \times (W_{n\times i}) = \mathrm{ESM}_{p\times i} \tag{1}$$

where W is the inverse of MM. Thus, for every estimated source *i* a vector $\boldsymbol{W}_{n\times 1}$ has to be estimated in such a way that the outputs of the matrix multiplication $(X'_{p\times n}) \times (\boldsymbol{W}_{n\times 1})$ are statistically independent of each other. The FastICA algorithm[11] is used to estimate these $\boldsymbol{W}_{n\times 1}$'s, which consists of the following steps:

a) Choose an initial random weight vector $\boldsymbol{W}_{n\times 1}$ of which the variance is 1.
b) Minimization of negentropy. The estimated sources have to be non-gaussian for estimation of the ICA model.[11] To use non-gaussianity in ICA model estimation, negentropy is introduced. Negentropy ($J(Y)$) of a random variable *Y* is defined using entropy function ($H(.)$) and a random variable following multivariate normal distribution (Y_{gauss}) as:

$$J(Y) = H(Y_{\text{gauss}}) - H(Y) \tag{2}$$

If the components are Gaussian, then the negentropy is zero. Non-gaussianity of the components increases if their negentropy increases. Therefore, negentropy is maximized to find components with maximum non-gaussiantity. Due to computational issues, negentropy is approximated as:

$$J(Y) \approx \left[E\big(G(Y)\big) - E\big(G(v)\big)\right]^2 \tag{3}$$

where $E(.)$ is expectation function of a random variable, v is a standard normal random variable and Y is standardized. For this analysis, the G function is chosen as:

$$G(u) = \log\cosh\,(u) \tag{4}$$

In the present study, non-gaussianity was measured by the approximation of negentropy $J(X'\boldsymbol{W})$ as described above.

c) Update $\boldsymbol{W}$:

1. $\boldsymbol{W}^{+} = E\{G'(X'\boldsymbol{W})X'\} - \boldsymbol{W}\,E\{G''(X'\boldsymbol{W})\}$ (5)

   i. Where G' and G'' are first and second derivatives of the function G.

2. $\boldsymbol{W} = {}^{\boldsymbol{W}^{+}}\!/_{//\boldsymbol{W}^{+}//}$ (6)

   ii. $//\boldsymbol{W}//$ represents norm of $\boldsymbol{W}$

d) If the norm of the difference between the old and new $\boldsymbol{W}$ is more than a tolerance level, then restart from step *b*. In the present study, the tolerance level was fixed at 0.0001.

e) If the old and new $\boldsymbol{W}$ are in the same direction with a fixed tolerance level, then the algorithm finds $X'\boldsymbol{W}$ as one ES.

f) To estimate several ESs together, run step *a* to *e* with different weight vectors and decorrelate the outputs ($X'\boldsymbol{W}$) after each iteration.

ICA was conducted using the fastICA function of the package fastICA version 1.2-0 in R version 3.3.1[11].

Consensus sources estimation on ICA

Due to maximization of negentropy in a large dimensional space, the FastICA algorithm can get stuck in the local maxima. The resulting ESs can be different for different initializations of the initial random weight matrix $W_{n\times i}$. The Consensus sources estimation (CSE) algorithm can be used to get a set of consensus estimated sources (CESs) for which the probability of negentropy converging to its local maxima is minimized. The assumption of CSE is that over a large number of runs of ICA, negentropy does not converge to any local maxima for most of the runs. In the present study, CSE was conducted on 25 different runs of ICA, each with a different random initialization of the weight matrix $W_{n\times i}$.[12] The CSE algorithm consists of the following steps:

a) Combine the $\mathrm{ESM}_{p\times i}$'s of all ICA runs together into a single matrix with p rows and $i\times$ number of ICA runs columns.
b) Clustering of highly correlated ESs. In the present study, ESs were clustered together when the absolute value of the Pearson correlation coefficient between them was > 0.9.
c) Computing CESs. For a cluster containing n ESs (ES_1, ES_2,…, ES_n), CESs are calculated as follows:

$$CES_{p\times 1} = (1/n)\sum_{i=1}^{n}(ES_i \times sign(correlation(ES_1, ES_i))) \quad (7)$$

The number of ESs in each cluster can be at most the total number of ICA runs (for the present study, that is 25). A credibility index is computed for each cluster as follows:

$$credibility\ index = \frac{number\ of\ ESs\ in\ that\ cluster}{total\ number\ of\ ICA\ runs} \quad (8)$$

The higher the credibility index, the higher the chance of obtaining the ESs for which the negentropy converges to its global maxima. In the present study, the cut-off for the credibility index was fixed at 50%. That is, clusters with a credibility index greater than 50% were only considered for obtaining the consensus estimated source matrix ($CESM$). The $CESM_{p\times m}$ contains $CES_{p\times 1}$'s from m clusters which had a credibility index greater than 50%. The characteristics of CESs are similar to those of ESs.

d) Computing consensus mixing matrix (CMM): - For the mRNA expression dataset $X_{p\times n}$ (The expression levels for each probeset (GEO, CCLE and GDSC) or gene (TCGA) were standardized to a mean of zero and variance of one to remove probeset-specific or gene-specific variability in the datasets) and $CESM_{p\times m}$, the $CMM_{m\times n}$ is obtained as follows:

$$CMM_{m\times n} = ((CESM')_{m\times p} \times CESM_{p\times m})^{-1} \times (CESM')_{m\times p} \times X_{p\times n} \tag{9}$$

where $CESM'$, is the transpose of $CESM$. The characteristics of the CMM are similar to those of the MM.

Detection of extreme-valued genomic region (DEGR)

We observed a pattern in many $CES_{p\times 1}$'s where specific contiguous genomic regions contain many probesets or genes with extreme-valued weights. We developed an algorithm called detection of extreme-valued genomic region (DEGR) to identify the contiguous genomic region(s) with statistically significant co-localization of extreme-valued weights in CESs. Using this algorithm, we quantified the number of CESs with this pattern. The DEGR algorithm consists of the following steps:

a) Collapsing probe-level weights of the $CESM$. The CESs identified in the GEO dataset, CCLE dataset and GDSC dataset contain probe-level weights. As multiple probesets can target a single gene, many genomic regions have a high chance of co-localizing extreme values corresponding to these probe-level weights. Therefore, probe-level weights need to be collapsed to gene-level weights. In the DEGR algorithm, out of multiple probe-level weights corresponding to the same gene, the probe-level weight with the highest absolute value is retained as gene-level weight.

b) Smoothing the $CESM$. To minimize the effect of outliers, smoothing is applied on the gene-level weights of CESs. Smoothing of the weights is performed per chromosome. If there are g number of genes in a single chromosome, then the smoothing coefficients C_{k1}, C_{k2}, …, C_{kg} are generated for the kth gene from the truncated normal distribution (TND). Next, the smoothing coefficients are updated in the following way:

$$C'_{ki=}C_{ki}/\sum_1^g C_{ki} \quad i = 1, 2, \dots, g \tag{10}$$

The smoothened weight of the kth gene in each CES (CES'_k) is obtained in the following way:

$$CES'_k = (CES_{g\times 1})' \times (C'_k)_{g\times 1} \tag{11}$$

where $CES_{g\times 1}$ is the subset of the CESs having weights from all genes mapping to that chromosome which contains the kth gene. For each gene, the mean of the TND is chosen as the base pair number of the gene. The standard deviation of the TND for genes mapping to the same chromosome is determined using the following steps:

1. Fix a set of possible standard deviation (sd) values as input. In the present study, input values were all integers from 10,000 to 2,000,000 with a gap of 10,000.
2. Calculate interval length (il) = 3 $\times$ sd
3. For every chromosome (ch)

    i. For every interval length (il)

    - For every gene (g) mapped to ch calculate the neighborhood density ($nd_{ch,g,il}$), where $nd_{ch,g,il}$ is the number of genes mapped to ch which have distance from the gene g in terms of base pair number $< il$.
    - Obtain 5% quantile ($quantile\_nd_{ch,g,il}$) of all the $nd_{ch,g,il}$'s for genes mapping to chromosome ch corresponding to interval length il.

    ii. Obtain the optimal interval length for chromosome ch (oil_{ch}), where oil_{ch} is the minimum of the il's for which $quantile\_nd_{ch,g,il} > 10$.

    iii. Assign the standard deviation parameter of the TND for genes mapping to chromosome ch as $oil_{ch}/3$.

c) Perform permutation test to mark extreme-valued weight indicator: Permutation test is pursued on each CES separately using the following steps.

1. 1000 permutation of the non-smoothened weights of the gene-level CES are retained in a matrix p_CESM_{px1000}.

2. Obtain smoothened permuted CESM (sp_CESM) using the smoothing method explained in the step *b*. Next, sort the absolute values of the weights of all the columns of sp_$CESM_{px1000}$ in decreasing order.
3. Sort the absolute values of the weights of the smoothened CES in decreasing order (sorted_CES).
4. For every column of sp_$CESM_{px1000}$ (sp_CES_i),
   i. For every weight of sorted_CES (sorted_CES_j), obtain the number of weights of sp_CES_i greater than sorted_CES_j ($f_{>j}$)
   ii. Obtain the optimal cutoff for the sp_CES_i (oc_i) as the maximum value of the weights of sorted_CES for which $f_{>j}/j$ > 5%.
5. Initial indicator marks (iim) for every weight of the smoothened CES (smoothened_CES_s) are obtained in the following way
   i. If smoothened_CES_s > median(oc) then iim_s = 1
   ii. If smoothened_CES_s < -median(oc) then iim_s = -1
   iii. Otherwise zero.
6. Smoothen iim (smoothened_iim) using the smoothing method described in step *b*.
7. Secondary indicator marks (sim) for every weight of the smoothened CES (smoothened_CES_s) are obtained in the following way:
   i. If smoothened_iim_s > 0.85 then sim_s = 1
   ii. If smoothened_iim_s < -0.85 then sim_s = -1
   iii. Otherwise zero.

8. Final indicator marks (fim) for every weight of the smoothened CES (smoothened_CES_s) are obtained in the following way

    i. Obtain the number of genes (ng_s) mapped to the corresponding chromosome which have a distance from gene s in terms of base pair number < corresponding optimal interval length (oil) as described in step *b.3.ii*.

    ii. If $ng_s < 10$ then $fim_s = 0$

    iii. Otherwise, $fim_s = sim_s$

d) Obtain the indicator matrix $\mathrm{IM}_{p\times m}$, where i^{th} column of $\mathrm{IM}_{p\times m}$ is fim corresponding to the ith CES. Hence, $\mathrm{IM}_{(k,i)}$ is 1 if the weight of kth gene of ith CES falls in an extreme-valued genomic region and zero otherwise.

Transcriptional adaptation to CNA profiling (TACNA profiling)

We hypothesized that the subset of CESs harboring a pattern in which contiguous genes are assigned extreme-valued weights might capture the degree of transcriptional adaptation to CNAs. These CESs are from now on referred to as CNA-CESs. To find support for this claim we first developed transcriptional adaptation to CNA profiling (TACNA profiling). In TACNA profiling, we reconstructed the gene expression profile for an individual sample by only using weights of extreme-valued genomic regions corresponding to the subset of CNA-CESs.

First, the matrix $\mathrm{TACNAP}_{p\mathrm{x}n}$ corresponding to TACNA profiles of *p* probesets/genes (for GEO, CCLE and GDSC, this analysis is done on probeset level and for TCGA, genelevel data is analyzed) and *n* samples is obtained using the following steps:

a) We obtain the indicator matrix $\mathrm{IM}_{p\times m}$ corresponding to *p* probesets/genes and *m* CESs after applying DEGR on CESM_{pxm}, where $\mathrm{IM}_{(k,i)}$ is 1 if the weight of the kth gene of the ith CES falls in extreme-valued genomic region and zero otherwise.

b) We obtain CNA-CESM_{pxm} as:

$$\text{CNA-CESM}_{(i,j)} = \mathrm{CESM}_{(i,j)} \mathrm{X}\ \mathrm{IM}_{(i,j)} \quad i = 1, 2, \ldots, p. j = 1, 2, \ldots, n \qquad (12)$$

c) We use the consensus mixing matrix $\mathrm{CMM}_{m\times n}$ corresponding to *m* CES's and *n* samples (step *d* of the CSE algorithm described above) to obtain initial TACNA profiles ($\text{initial\_TACNAP}_{pxn}$) in the following way:

$$\text{initial\_TACNAP}_{p\times n} = \mathrm{CNA} - \mathrm{CESM}_{p\times m} \times \mathrm{CMM}_{m\times n} \qquad (13)$$

d) Consensus-ICA on probelevel / genelevel standardized expression data always leads to specific consensus estimated sources and mixing matrix which re-generate TACNA profiles with average expression value of zero for each probeset / gene. The value zero in the re-generated initial TACNA profiles corresponds to transcriptional adaptation to average copy number alteration of all the samples. It is well studied that almost all types of tumors frequently have genomic alterations with gain or loss of the whole or parts of chromosomes. Hence, the ploidy of the tumor cells cannot be assumed to be 2n. As the majority of samples in the GEO dataset and TCGA dataset represent tumor tissue, the average ploidy in these datasets are not 2n. To ensure zero weights in initial TACNA profiles correspond to 2n ploidy, we adjusted the initial TACNA profiles from the GEO dataset and TCGA dataset with robust mean (Hodges Lehmann estimate) expression of the normal samples in the corresponding datasets for every probeset / gene[13]. The profiles after the above adjustment are defined as TACNA profiles.

$$\text{TACNAP}_{p\times n} = \text{initial\_TACNAP}_{p\times n} - \text{HodgesLehmannEstimate}(\text{initial\_TACNAP}_{\text{normal}}) \quad (14)$$

Transcriptional adaptation to CNA with CNA-CES's having 50 or more genes in extreme valued region profiling (TACNA50 profiling)

In many of the analyses presented in our manuscript, we used CNA-CESs having 50 or more genes in extreme valued region for obtaining TACNA profiles (TACNA50 profiling). This very stringent threshold for TANCA50 profiling was used to ensure that any biological association found with the degree of transcriptional adaptation of a gene was not the result of false positive findings, which could be the result of a CESs being incorrectly labeled as a CNA-CES by the DEGR algorithm. On the other hand, the change of false negative associations increased, but we deemed this in the context of this manuscript less harmful. The matrix $TACNA50P_{pxn}$ corresponding to TACNA50 profiles of *p* probesets/genes (for GEO, CCLE and GDSC, this analysis is done on probeset level and for TCGA, genelevel data is analyzed) and *n* samples is obtained using the steps for generating the initial TACNA profiles only with an updated IM_{pxm} ($IM50_{pxm}$). The steps to obtain $IM50_{pxm}$ are given below:

a) We obtain the indicator matrix $\text{IM}_{p\times m}$ corresponding to *p* probesets/genes and *m* CES's after applying DEGR on $CESM_{pxm}$, where $IM_{(k,i)}$ is 1 if the weight of the kth gene of the ith CES falls in extreme-valued genomic region and zero otherwise.

b) We obtain a vector $ngevr_{mx1}$ (number of genes in EVR) for each column of IM_{pxm} using the following steps:

   1. If IM_{pxm} is on probeset level, then convert it to genelevel using step 'a' of DEGR algorithm.

2. Then for each column i,

   i. $\text{ngevr}(i) = \text{sum}(\text{IM}_{(,i)})$ (15)

c) A new matrix IM50_{pxm} is generated in the following way:

   1. All entries of ith column of IM50_{pxm} ($\text{IM50}_{(,i)}$) = 0 for all i's where ngevr(i) < 50

   2. $\text{IM50}_{(,i)} = \text{IM}_{(,i)}$ for all i's where ngevr(i) >= 50

Cross-validation analysis within one platform

A cross-validation analysis of TACNA-profiling was conducted to test the robustness of this method within one platform. A five-fold cross-validation analysis an mRNA expression dataset was done using the following steps:

a) Samples from the mRNA expression dataset were randomly divided into five groups using a multinomial distribution simulation.

b) Gene expression profiles from the mRNA expression dataset were standardized on the gene-level to a mean of zero and a standard deviation of one (standardized_mRNA).

c) For the ith fold the following steps were conducted (i = 1,2,3,4 and 5):

   a. The input dataset (mRNA_CV_i) for next steps was obtained by excluding samples in the ith group from the unstandardized mRNA expression dataset.

   b. Gene expression profiles from mRNA_CV_i were standardized to a mean of zero and standard deviation of one.

   c. Consensus independent component analysis was applied on the standardized mRNA_CV_i to obtain consensus estimated sources matrix (CESM_i).

d. CESM_i and $\mathrm{standardized\_mRNA}$ were used to obtain the consensus mixing matrix (CMM_i):

e. $\mathrm{CMM}_i = ((\mathrm{CESM}_i)' \times \mathrm{CESM}_i)^{-1} \times (\mathrm{CESM}_i)' \times \mathrm{standardized\_mRNA}$ (16)

f. CESM_i with extreme valued contiguous genomic regions ($\mathrm{CNA\_CESM}_i$) were identified using the DEGR algorithm as described in the Supplementary Note.

g. $\mathrm{CNA\_CESM}_i$ and the CMM_i were used to obtain TACNA profiles for the samples present in $\mathrm{mRNA\_CV}_i$ along with the samples in the ith group ($\mathrm{TACNAP\_excluded}_i$).

Pearson correlation coefficients were calculated between $\mathrm{TACNAP\_excluded}_i$ and corresponding CNA profiles (derived from SNP arrays).

Cross-study cross-validation analysis

A cross-study cross-validation analysis of TACNA-profiling was conducted to test the robustness of this method across different studies or platforms. Following steps were conducted for each cross-study cross-validation analysis where consensus estimated sources of dataset i were used to obtain TACNA-profiles of dataset j:

d) Genes not present in both dataset i and j were removed from the analysis.

e) Both dataset i and dataset j were standardized on gene-level separately, which means each gene expression is transformed to a mean of zero and standard deviation of one.

f) Both of these standardized datasets were sample-wise merged to obtain a combined dataset ($\mathrm{Combined\_i\_used\_for\_j}$).

g) Consensus estimated sources matrix of dataset i (CESM_i) and $\mathrm{Combined\_i\_used\_for\_j}$ were used to obtain consensus mixing matrix ($\mathrm{CMMcombined\_i\_used\_for\_j}$).

$$\mathrm{CMMcombined\_i\_used\_for\_j} = ((\mathrm{CESM}_i)' \times \mathrm{CESM}_i)^{-1} \times (\mathrm{CESM}_i)' \times \mathrm{Combined\_i\_used\_for\_j} \quad (17)$$

h) $\mathrm{CESM_i}$ with extreme valued contiguous genomic region ($\mathrm{CNA\_CESM}_i$) were identified using DEGR algorithm.

i) $\mathrm{CNA\_CESM}_i$ and $\mathrm{CMMcombined\_i\_used\_for\_j}$ were used to obtain TACNA-profiles for the samples present in dataset i along with the samples in the dataset j ($\mathrm{TACNAP\_j\_using\_i}$).

j) Pearson correlation coefficient between $\mathrm{TACNAP\_j\_using\_i}$ and corresponding copy number profiles (derived from SNP arrays) were obtained.

Cross-dataset heterogeneity in CNA occurrence is a constraint to reconstruct TACNA profiles of a dataset using $\mathrm{CNA\_CESM}$ of any other dataset.

Human fragile sites

Previously described genomic locations of aphidicolin-induced fragile sites identified through cytogenic analyses were used to assess the colocalization of borders of marked genomic regions in CNA-CESs with common fragile sites.[14] Genomic coordinates were converted to human genome reference GRCh38 using the Batch Coordinate Conversion tool from USCS Genome Browser.[15] Borders of marked genomic regions in CNA-CESs were assumed to colocalize with common fragile sites when one or both borders were located in a common fragile site.

One-to-one gene- "best probeset" mapping for Affymetrix human microarrays

As multiple probesets can target a single gene on Affymetrix gene expression microarrays, it can present a mild conundrum while attempting to obtain the enrichment score of a 'gene-set' defined by gene names rather than corresponding names of probesets. The R package 'jetset' (version 3.4.0) was used to obtain one-to-one mapping between genes and the 'best' probesets for expression data generated with Affymetrix HG-U133 Plus 2.0 and Affymetrix Human Genome U219.[16] This package computes three scores for each probeset and then obtains an overall score to determine the best probeset for corresponding gene:

a) Specificity score: - the specificity score is defined as the fraction of probes in a probe set that are likely to detect the targeted gene and unlikely to detect other genes.
b) Coverage score: - the fraction of splice isoforms belonging to the targeted gene that are detected by the probeset is defined as coverage score of the probeset.
c) Robustness score: - the robustness score quantifies robustness against transcript degradation.

The overall score corresponding to each probeset is the product of the specificity score, coverage score, and robustness score. The probeset with the highest overall score is considered as the "best probeset" corresponding to the targeting gene and used in subsequent analyses.

Gene Set Enrichment Analysis

GSEA was performed utilizing 12 gene set databases from the MSigDB.[17] Gene sets containing less than 10 genes or more than 500 genes (after filtering out genes that were not present in our

data sets) were excluded from further analysis. Enrichment of a gene set was tested according to the two-sample Welch's t-test for unequal variance.

To obtain the metrics on which GSEA needed to be performed, we transformed the weights of the genes in each CNA-CES separately to metrics ranging from zero to one. Here, a metric of zero would correspond to the highest absolute weight (i.e., low degree of transcriptional adaptation to CNAs). A proportion of genes (38% of all genes in the GEO dataset and 25% of all genes in the TCGA dataset) had multiple metrics as they appeared in extreme-valued regions of multiple CNA-CESs. The lowest metric corresponding to these genes were considered for this analysis.

Welch's t-test was conducted between the set of metrics of genes whose corresponding gene identifiers are members of the gene set under investigation and the set of metrics of genes whose corresponding gene identifiers are not members of the gene set under investigation. To be able to compare gene sets of different sizes, Welch's t statistics were transformed to $-\log_{10}$(P-value). To control the false discovery rate, we performed a multivariate permutation test with 100 permutations. For each permutation round gene identifiers were randomly assigned to metrics. This allowed us to present the number of significantly enriched gene sets per gene set database using a false discovery rate of 1% and a confidence level of 80%.

Inferred CNA burden

For each sample in the GEO dataset and TCGA dataset, CNA load was estimated as the sum of the 'coefficients' (i.e. indirect activity measurements) of all CNA-CESs in the MM with at least 50

genes in their marked genomic regions. CNA loads were normalized to a 0-1 range across samples of the same tumor type in each dataset separately.

Expression-based immune metric

A previously defined set of genes describing CD8+ T cell and natural killer cell activity (*CD2*, *CD3E*, *CD247*, *GZMK*, *NKG7* and *PRF1*) was used to calculate immune metric scores[18]. Per sample, the rank position of the mRNA expression levels of each of these genes was calculated. Scores for each sample were determined by calculating the mean rank position of the seven genes. Immune metric scores were normalized to a 0-1 range across samples of the same tumor type in each dataset separately.

Estimating immune cell type abundance

Immune cell type abundance was estimated using CIBERSORT.[19] Earlier, we hypothesized that each CES describes the effect of a transcriptional regulatory factor on gene expression levels. As CNAs generally do not occur in non-tumor tissue, we reconstructed gene expression profiles using all CESs but CNA-CESs (i.e. residual profiling) to more accurately capture the effects from the tumor microenvironment on gene expression levels. Next, the abundance of 22 immune cell types were estimated by applying the leukocyte gene signature matrix (LM22) on the residual profiles.

Prediction of gene functionalities

We used a GBA approach to predict likely functions for genes based on gene co-regulation. For this, we conducted a consensus-ICA on an unprecedented scale (manuscript in preparation). In short, a covariance matrix was calculated between 19,635 genes using the expression patterns of 106,462 gene expression profiles generated with Affymetrix HG-U133 Plus 2.0 representing the many disease states, cellular states, and genetic and chemical perturbations that were obtained. Consensus-ICA was performed on the covariance matrix, which resulted in the identification of a large set of CESs and a mixing matrix reflecting the activity of each source in the expression pattern of each gene across the samples. Next, a GBA approach was used to predict the functionality of individual genes. First, we retrieved 16 public gene set collections describing a large range of biological processes and phenotypes. For each gene set, we calculated its 'bar code' by averaging the MM weight of its member genes. Next, for each gene in the MM, the distance correlation was determined between its MM weights and the gene set bar code. A high correlation between a gene's MM weight and a gene set bar code indicated that the gene under investigation shared a functionality with the genes of the specific gene set under investigation. Significance levels were obtained with permutated data (250 permutations). This strategy was used on 23,372 well-described functional gene sets, which enabled us to create a comprehensive network of predicted functionalities of individual genes. This framework is available at http://www.genetica-network.com.

Protein complexes analysis

CORUM complex gene sets were collected from CORUM website ‘https://mips.helmholtz-muenchen.de/corum/#download’ (Core complex set). Complexes not mapping to ‘Human’ organism were discarded. Genes that had a ‘None’ value in the ‘subunits(Entrez IDs)’ column were discarded, which correspond to genes that have a UNIPROTID but no Entrez ID. Finally, protein complex gene sets with 4 or less subunits were discarded. Pearson correlation matrices for each protein complex (n = 304) were calculated using TACNA50 expression levels from the TCGA dataset (n = 10,817). For each correlation matrix, a density metric was defined as the median value of the correlation matrix after discarding the diagonal. For each protein complex, 1,000 similarly sized random groups of genes were generated and their density metric was calculated. A P value was assigned to each complex by calculating the fraction of permutations with lower density metrics as the real protein complex.

Association between DNA methylation and degree of transcriptional adaptation

For a subset of samples in the TCGA dataset (n = 9,317) available preprocessed methylation data generated with the Illumina 450 K array was collected. For each sample, we obtained the β-values of all individual genes. These β-values for individual genes were calculated using the mean signal values of methylation probes mapping to the same gene. In other words, β-values resemble the mean methylation level of individual genes in a given sample. For each gene, we correlated its mean methylation levels with TACNA expression levels across all samples.

Identification of CES capturing gender differences

Biological gender annotation for a subset of samples from the GEO dataset was collected from the GEO portal. A Mann-Whitney U test was performed for every CES comparing the mixing matrix weights of samples annotated to be male and samples annotated to be female. CES 471 had the highest discrimination power between male and female samples (AUC = 0.9867).

Expression quantitative trait loci (eQTL) analyses

eQTL analysis was conducted using the mRNA expression profiles, the TACNA profiles and the CNA profiles of the TCGA dataset (n = 10,817). Details are given below:

- Genes and samples not present in all three of the above-mentioned datasets were removed from the analysis.
- Pearson correlation coefficients were obtained between mRNA expression profiles and CNA profiles on the gene-level.
- Pearson correlation coefficients were obtained between TACNA profiles and CNA profiles on the gene-level.
- Association within the CNA profiles on the gene-level was computed using Pearson correlation coefficient.

Partial correlation coefficients between TACNA profiles and CNA profiles were computed on the gene-level to identify trans effect. Partial correlation analysis was conducted to remove false positive trans effects driven by CNA co-occurrence.

TACNA profile versus mRNA changes upon chromosome 5 transfer

Expression profiles were downloaded from the Gene Expression Omnibus for samples GSM978891-96 belonging to series GSE39768 performed on platform GPL4133. In addition,

2,949 additional samples were downloaded for other studies performed on the same platform which had also Cy3 single label data available. All genes in this joint GPL4133 dataset were then median centered and log_2 transformed. TACNA profiles were generated for the GPL4133 dataset using the sources obtained for the TCGA dataset (see Cross-study cross-validation section of Supplementary Note). To calculate arithmetic differences between the two conditions an average was calculated for the control arm (GSM978891-93) and another for the experimental arm (GSM978894-96) of the HCT116 model of chromosome 5 tetrasomy performed in GSE39768. Pearson correlations were calculated between the differences in mRNA expression and the differences of TACNA profiles using only genes mapping to chromosome 5.

References

1. Barrett T, Wilhite SE, Ledoux P, et al. NCBI GEO: archive for functional genomics data sets — update. Nucleic Acids Res. 2013;41:991–995.

2. Li B, Dewey CN. RSEM: accurate transcript quantification from RNA-Seq data with or without a reference genome. BMC Bioinformatics. 2011;12:323.

3. NCI Genomic Data Commons (GDC). GDC data user's guide. Available at: https://docs.gdc.cancer.gov/Data/PDF/ Data_UG.pdf (2018).

4. Korn JM, Kuruvilla FG, McCarroll SA, et al. Integrated genotype calling and association analysis of SNPs, common copy number polymorphisms and rare CNVs. Nat Genet. 2008;40:1253–1260.

5. Olshen AB, Venkatraman ES, Lucito R, Wigler M. Circular binary segmentation for the analysis of array-based DNA copy number data. Biostatistics. 2004;5:557–572.

6. Barretina J, Caponigro G, Stransky N, et al. The Cancer Cell Line Encyclopedia enables predictive modelling of anticancer drug sensitivity. Nature. 2012;483:603–307.

7. Iorio F, Knijnenburg TA, Vis DJ, et al. A landscape of pharmacogenomic interactions in cancer. Cell. 2016;166:740–754.

8. Greenman CD, Bignell G, Butler A, et al. PICNIC: An algorithm to predict absolute allelic copy number variation with microarray cancer data. Biostatistics. 2010;11:164–175.

9. Bolstad BM, Irizarry RA, Astrand M, Speed TP. A comparison of normalization methods for high density oligonucleotide array data based on variance and bias. Bioinformatics. 2003;19:185–193.

10. Comon P. Independent component analysis, a new concept? Signal Processing. 1994;36:287–314.

11. Hyvärinen A, Oja E. Independent component analysis: algorithms and applications. Neural Networks. 2000;13:411–430.

12. Chiappetta P, Roubaud MC, Torrésani B. Blind source separation and the analysis of microarray data. J Comput Biol. 2004;11:1090–1109.

13. Hershberger SL. Hodges-Lehmann Estimators. In: International Encyclopedia of Statistical Science (ed. Lovric, M) pp 635–636 (Springer Berlin Heidelberg, 2011).

14. Fungtammasan A, Walsh E, Chiaromonte F, Eckert KA, Makova KD. A genome-wide analysis of common fragile sites: what features determine chromosomal instability in the human genome ? Genome Res. 2012;22:993–1005.

15. Casper J, Zweig AS, Villarreal C, et al. The UCSC Genome Browser database: 2018 update. Nucleic Acids Res. 2018;46:D762–D769.

16. Li Q, Birkbak NJ, Gyorffy B, Szallasi Z, Eklund AC. Jetset: selecting the optimal microarray probe set to represent a gene. BMC Bioinformatics. 2011;12:474.

17. Liberzon A, Birger C, Thorvaldsdóttir H, et al. The Molecular Signatures Database hallmark gene set collection. Cell Syst. 2015;1:417–425.

18. Rooney MS, Shukla SA, Wu CJ, Getz G, Hacohen N. Molecular and genetic properties of tumors associated with local immune cytolytic activity. Cell. 2015;160:48–61.

19. Newman AM, Liu CL, Green MR, et al. Robust enumeration of cell subsets from tissue expression profiles. Nat Methods. 2015;12:453–457.
