# Supplementary material for: Transcriptional effects of copy number alterations in a large set of human cancers
Source: Nat Commun. 2020 Feb 5;11:715. doi: 10.1038/s41467-020-14605-5 (PMC7002723; doi:10.1038/s41467-020-14605-5)
Supplement: Supplementary file 21 — Reporting Summary [file 41467_2020_14605_MOESM21_ESM.pdf]

nature research

Corresponding author(s): Rudolf S.N. Fehrmann

Last updated by author(s): Jan 7, 2020

Reporting Summary

Nature Research wishes to improve the reproducibility of the work that we publish. This form provides structure for consistency and transparency in reporting. For further information on Nature Research policies, see Authors & Referees and the Editorial Policy Checklist.

Statistics

For all statistical analyses, confirm that the following items are present in the figure legend, table legend, main text, or Methods section.

n/a	Confirmed	
☐	☒	The exact sample size (*n*) for each experimental group/condition, given as a discrete number and unit of measurement
☐	☒	A statement on whether measurements were taken from distinct samples or whether the same sample was measured repeatedly
☐	☒	The statistical test(s) used AND whether they are one- or two-sided<br>*Only common tests should be described solely by name; describe more complex techniques in the Methods section.*
☐	☒	A description of all covariates tested
☐	☒	A description of any assumptions or corrections, such as tests of normality and adjustment for multiple comparisons
☐	☒	A full description of the statistical parameters including central tendency (e.g. means) or other basic estimates (e.g. regression coefficient) AND variation (e.g. standard deviation) or associated estimates of uncertainty (e.g. confidence intervals)
☐	☒	For null hypothesis testing, the test statistic (e.g. *F*, *t*, *r*) with confidence intervals, effect sizes, degrees of freedom and *P* value noted<br>*Give P values as exact values whenever suitable.*
☐	☒	For Bayesian analysis, information on the choice of priors and Markov chain Monte Carlo settings
☐	☒	For hierarchical and complex designs, identification of the appropriate level for tests and full reporting of outcomes
☐	☒	Estimates of effect sizes (e.g. Cohen's *d*, Pearson's *r*), indicating how they were calculated

Our web collection on statistics for biologists contains articles on many of the points above.

Software and code

Policy information about availability of computer code

Data collection

Microarray expression data was collected from three public data repositories: Gene Expression Omnibus with accession number GPL570 (generated with Affymetrix HG-U133 Plus 2.0), CCLE (generated with Affymetrix HG-U133 Plus 2.0, file CCLE_Expression.Arrays_2013-03-18.tar.gz) available at https://portals.broadinstitute.org/ccle/data and GDSC (generated with Affymetrix HG-U219) available at https://www.ebi.ac.uk/arrayexpress/experiments/E-MTAB-3610/. Preprocessing and aggregation of raw expression data was performed using the robust multi-array average algorithm with RMAExpress (version 1.1.0)37. Quality control of the processed expression data is described in the Supplementary Note. Pre-processed and normalized RNA-seq data was collected from TCGA using the Broad GDAC Firehose portal (https://gdac.broadinstitute.org/). In each dataset, expression levels for every gene were standardized to a mean of zero and variance of one. In addition, CNA profiles generated with Affymetrix Genome-Wide Human SNP Array 6.0 were collected for a subset of samples in the TCGA dataset, CCLE dataset and GDSC dataset and processed as described in Supplementary Note.

Softwares used: CIBERSORT, R version 3.3.1, package fastICA version 1.2-0, data.table version 1.12.8, sqldf version n 0.4-11, sva version 3.34.0, evd version 2.3-3, rARPACK version 0.11-0, DBI version 1.1.0, caret version 6.0-84, gPCA version 1.0, ConsensusClusterPlus version 1.50.0, foreach version 1.4.7, doMC version 1.3.6, biganalytics version 1.1.14, clValid version 0.6-6, amap version 0.8-18, sendmailR version 1.2-1, truncnorm version 1.0-8, parallel part of R 3.6.2.

Data analysis

Custom code was extensively used and is made available at the website. http://www.genomicinstability.org/ -> Download support data -> Source code -> Source_codes.zip

Also the codes are available at https://github.com/arkajyotibhattacharya/TACNA_profiling.

For manuscripts utilizing custom algorithms or software that are central to the research but not yet described in published literature, software must be made available to editors/reviewers. We strongly encourage code deposition in a community repository (e.g. GitHub). See the Nature Research guidelines for submitting code & software for further information.

Data

Policy information about availability of data

All manuscripts must include a data availability statement. This statement should provide the following information, where applicable:

- Accession codes, unique identifiers, or web links for publicly available datasets
- A list of figures that have associated raw data
- A description of any restrictions on data availability

The datasets generated during and/or analysed during the current study are available in the website http://www.genomicinstability.org/. TACNA gene distribution and degree of TACNA can be explored at the gene level in top four panels of the above website. Additionally, the tab Download support data in the website links to zip files that contain TACNA profiles and degree of TACNA estimates for all four datasets.

Field-specific reporting

Please select the one below that is the best fit for your research. If you are not sure, read the appropriate sections before making your selection.

☒ Life sciences ☐ Behavioural & social sciences ☐ Ecological, evolutionary & environmental sciences

For a reference copy of the document with all sections, see nature.com/documents/nr-reporting-summary-flat.pdf

Life sciences study design

All studies must disclose on these points even when the disclosure is negative.

Sample size	Our study is a reanalysis of publicly available gene expression profiles and therefore utilizes the data made available. We downloaded all available samples at the time of analysis.
Data exclusions	Samples that were determined to be duplicates were excluded from analysis. Samples that did not pass our quality control were also excluded. Quality control was performed according to previously described methodology. For quality control, principal component analysis (PCA) was applied on the sample Pearson product-moment correlation matrix. The first principal component (PCqc) of such an expression microarray correlation matrix describes nearly always a constant pattern that dominates the data, explaining around 80-90% of the total variance. This pattern can be regarded as probe-specific or platform-specific variance, independent of the biological sample hybridized to the array. The correlation of each individual microarray expression profile with this PCqc (in PCA analysis called factor loadings) can be used to detect outliers, as arrays of lesser quality will have a lower correlation with the PCqc. We removed samples with a Pearson $r < 0.8$. Because individual samples could be uploaded multiple times to repositories, we checked our datasets for duplicates. Duplicate CEL files were removed by generating a message-digest algorithm 5 (MD5) hash for each individual CEL file. A MD5 hash acts like a unique fingerprint for each individual file and duplicate CEL files will have an identical MD5 hash.
Replication	We performed TACNA profiling on 4 diferent datasets wich contain samples collected from patient material and cell lines using microarray or RNA-seq to show that our method is reproducible.
Randomization	Randomization was performed for cross-validation analyses. For cross-validation analysis in each dataset, 20% of samples were randomly selected for which TACNA profiles were generated using CNA-CESs identified in the remaining 80% of samples.
Blinding	Blinding was not performed but the method was validated using a 5-fold study cross-validation and 5-fold cross-study cross-validation strategy.

Reporting for specific materials, systems and methods

We require information from authors about some types of materials, experimental systems and methods used in many studies. Here, indicate whether each material, system or method listed is relevant to your study. If you are not sure if a list item applies to your research, read the appropriate section before selecting a response.

Materials & experimental systems

n/a	Involved in the study
☒	☐ Antibodies
☒	☐ Eukaryotic cell lines
☒	☐ Palaeontology
☒	☐ Animals and other organisms
☒	☐ Human research participants
☒	☐ Clinical data

Methods

n/a	Involved in the study
☒	☐ ChIP-seq
☒	☐ Flow cytometry
☒	☐ MRI-based neuroimaging
